# 3-chymotrypsin-like protease in SARS-CoV-2

**DOI:** 10.1042/BSR20231395

**Published:** 2024-08-05

**Authors:** Kenana Al Adem, Juliana C. Ferreira, Adrian J. Villanueva, Samar Fadl, Farah El-Sadaany, Imen Masmoudi, Yugmee Gidiya, Tariro Gurudza, Thyago H.S. Cardoso, Nitin K. Saksena, Wael M. Rabeh

**Affiliations:** 1 Science Division, New York University Abu Dhabi, PO Box 129188, Abu Dhabi, United Arab Emirates; 2 OMICS Centre of Excellence, G42 Healthcare, Masdar City, Abu Dhabi, United Arab Emirates; 3 Victoria University, Footscray Campus, Melbourne, VIC. Australia

**Keywords:** 3-chymotrypsin-like protease (3CLpro), Covalent inhibitors, COVID-19, main protease, Non-covalent inhibitors, SARS-CoV-2

## Abstract

Coronaviruses constitute a significant threat to the human population. Severe acute respiratory syndrome coronavirus-2, SARS-CoV-2, is a highly pathogenic human coronavirus that has caused the coronavirus disease 2019 (COVID-19) pandemic. It has led to a global viral outbreak with an exceptional spread and a high death toll, highlighting the need for effective antiviral strategies. 3-Chymotrypsin-like protease (3CLpro), the main protease in SARS-CoV-2, plays an indispensable role in the SARS-CoV-2 viral life cycle by cleaving the viral polyprotein to produce 11 individual non-structural proteins necessary for viral replication. 3CLpro is one of two proteases that function to produce new viral particles. It is a highly conserved cysteine protease with identical structural folds in all known human coronaviruses. Inhibitors binding with high affinity to 3CLpro will prevent the cleavage of viral polyproteins, thus impeding viral replication. Multiple strategies have been implemented to screen for inhibitors against 3CLpro, including peptide-like and small molecule inhibitors that covalently and non-covalently bind the active site, respectively. In addition, allosteric sites of 3CLpro have been identified to screen for small molecules that could make non-competitive inhibitors of 3CLpro. In essence, this review serves as a comprehensive guide to understanding the structural intricacies and functional dynamics of 3CLpro, emphasizing key findings that elucidate its role as the main protease of SARS-CoV-2. Notably, the review is a critical resource in recognizing the advancements in identifying and developing 3CLpro inhibitors as effective antiviral strategies against COVID-19, some of which are already approved for clinical use in COVID-19 patients.

## Introduction

To date, seven different coronaviruses have been identified to infect humans, three of which have emerged in the last 20 years, causing serious viral epidemics, including the severe acute respiratory syndrome coronavirus (SARS-CoV), the Middle East respiratory syndrome coronavirus (MERS-CoV) and severe acute respiratory syndrome coronavirus-2 (SARS-CoV-2) [1]. Most recently, SARS-CoV-2 caused the coronavirus disease 2019 (COVID-19) pandemic that first emerged in late 2019 in Wuhan, China [2–4]. Like other viruses, SARS-CoV-2 continues to evolve and accumulate several mutations in its viral genome since the beginning of the pandemic [5,6]. Based on the World Health Organization, several SARS-CoV-2 variants of concern have emerged that are characterized by higher transmissibility and virulence, including α, β, γ, δ, and omicron, the last of which has still been circulating worldwide for more than a year [5,7,8]. The COVID-19 pandemic has led to a global viral outbreak with an exceptional spread and a high death toll, highlighting the need for effective antiviral therapeutics. Transmission of COVID-19 is reported to occur through inhalation of infected droplets and aerosols, which can lead to clinical manifestations of respiratory illness [9–12]. While some cases are asymptomatic, most cases present with common symptoms such as fever, cough, sore throat, and shortness of breath and may progress to severe illnesses such as pneumonia and lung failure [13–19]. SARS-CoV-2 was also reported to distress other organs such as the gastrointestinal tract, kidneys, endocrine glands, and the central nervous system [20,21].

Coronaviruses (CoVs) constitute the largest family in the order of Nidovirales and are further divided into four genera, including α-, β-, γ-, δ-, and Δ-CoVs [1,22]. α- and β-CoVs are reported to infect several mammalian species, including humans, rodents, camels, cats, and bats, whereas gamma and delta CoVs are reported to infect birds [22]. While the human beta CoVs, SARS-CoV, MERS-CoV, and SARS-CoV-2, have been known to cause fatal viral outbreaks, four other endemic human CoVs are known to cause episodes of the common cold worldwide, including the NL63-CoV and 229E-CoV (α-CoVs) as well as OC43-CoV and HKU1-CoV (β-CoVs) [23–27].

**Figure 1:**
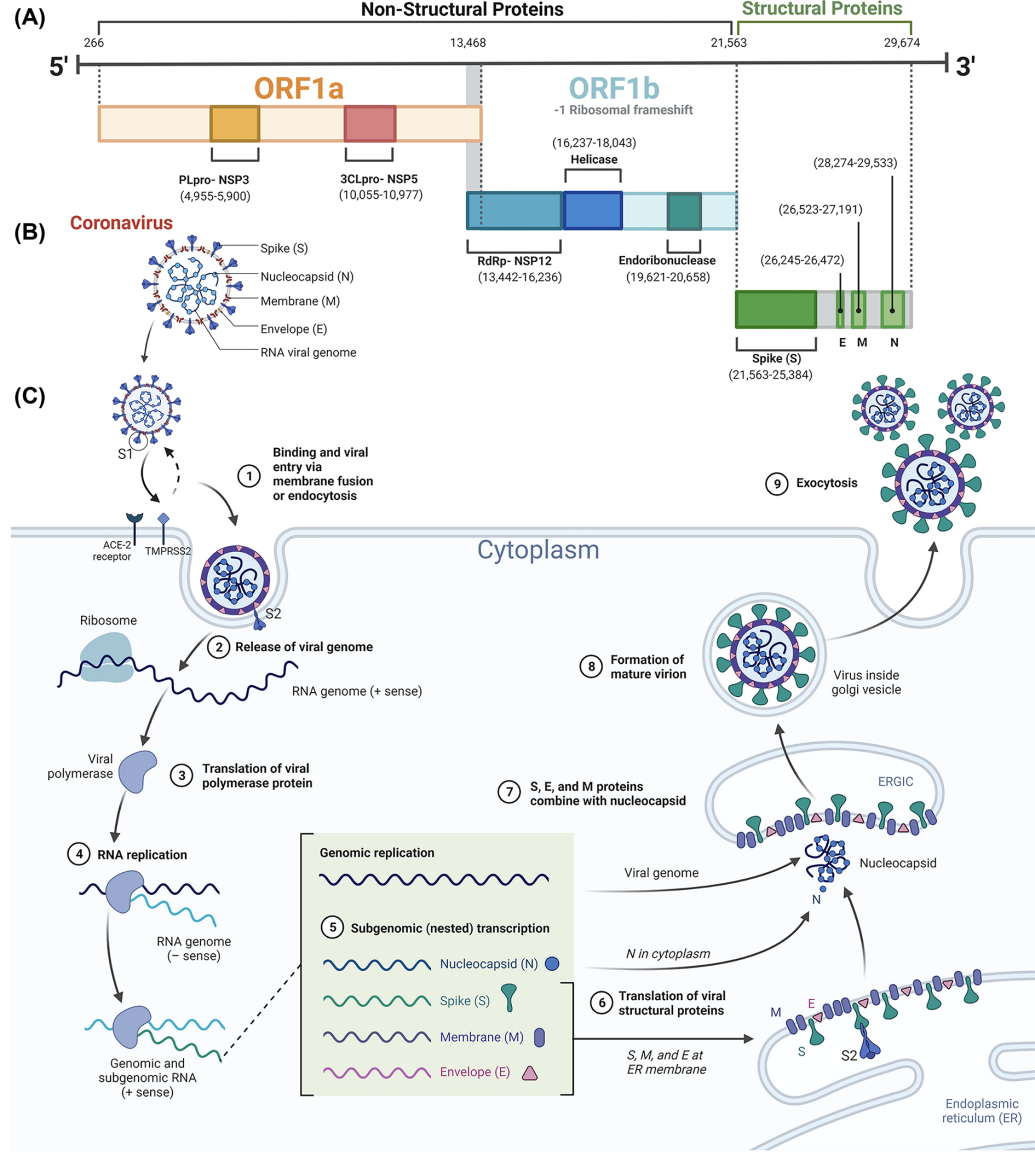
Genome, structure, and life cycle of SARS-CoV-2 (**A**) Genomic organization featuring functional domains depicted in rectangles characterizes the SARS-CoV-2 genome. The initial segment of the genomic RNA encodes NSPs, which undergo direct translation into two polyproteins, pp1a and pp1ab. A frameshift between ORF1a and ORF1b introduces variations in translation. Viral proteases, including PLpro and 3CLpro, cleave the polyproteins to yield 16 NSPs, forming the basis for the replication and transcription machinery. The critical functional domains within each NSP are illustrated in the genomic structure. The latter part of the RNA genome predominantly codes for four essential structural proteins and accessory proteins. (**B**) SARS-CoV-2 viral particle contains the structural proteins: the spike S-protein, membrane M-protein, envelope E-protein, and nucleocapsid N-protein. (**C**) The life cycle of SARS-CoV-2, including viral entry, replication and transcription, assembly, and release. The graphical representation illustrates the sequence of events, starting from host cell recognition and progressing through the release of new virions, depicted as steps 1 to 9.

All CoVs are enveloped spherical assemblies (∼125 nm) that contain a single-stranded positive-sense RNA genome (>30 kb) [28]. Under the microscope, CoVs have club-shaped protrusions that look like solar corona (corona refers to the Latin name for crown). These protrusions correspond to the viral spike protein that enables viral entry into host cells [29–31]. CoVs feature a conserved genomic organization that encodes nonstructural, structural, and accessory proteins (Figure 1A) [28]. Genome analysis showed that non-structural proteins are more conserved (58% similarity) than structural proteins (43% similarity), with the latter requiring greater diversity to adapt to different hosts [28]. The genome of SARS-CoV-2 comprises 15 open reading frames (ORFs), with one-third encoding the spike (S), envelope (E), membrane (M), and nucleocapsid (N) structural proteins (Figure 1B). Structural viral proteins hold essential roles such as viral RNA protection (N protein) [32,33], viral entry mechanism into host cells (S protein), virus production and maturation (E protein) [34–36], and membrane formation of new viral particles (M protein) [36,37].

SARS-CoV-2 S-protein is a trimeric glycoprotein with other human viruses having similar trimeric glycoprotein like influenza hemagglutinin, paramyxovirus fusion protein (F), Ebola virus glycoprotein, and HIV envelope glycoprotein (Env) [38]. The polysaccharide molecules envelop the viral spikes and act as camouflage to evade detection by the human immune system during the infectious phase [39]. The Spike (S) protein comprises two subunits: S1 and S2 [30]. During viral infection, SARS-CoV-2 S-protein targets the angiotensin-converting enzyme 2 (ACE2) as the host receptor [40]. ACE2 receptors are prevalent in various tissues, including the heart, kidneys, intestine, and pulmonary tissues [41]. Notably, the alveolar epithelial type II cells (AECII) are responsible for approximately 83% of ACE2 expression, emphasizing the pivotal role of these cells as the primary target for SARS-CoV-2 viral infections [42]. In particular, the S protein is a key element for the entry of SARS-CoV-2 into host cells (Figure 1C) and has been extensively studied in literature as a target for all vaccines and other anti-viral drugs against COVID-19 [8,29–31,43–53].

The remaining two-thirds of the SARS-CoV-2 genome is made up of ORF1a and ORF1b, which get immediately translated by the host to generate two overlapping long polyproteins, pp1a, and pp1ab containing the nonstructural proteins (nsps), nsp1-11 and nsp12-16, respectively [22,54,55]. Nsps play various roles in the viral life cycle, such as viral RNA synthesis and processing, proteolysis of the viral polyproteins, host mRNA degradation, host immune response suppression, and formation of viral double-membrane vesicles (Figure 1C) [55–57]. Several nsps have indispensable roles in SARS-CoV-2 replication and are recognized as main drug targets for antiviral therapies for COVID-19. Nsp12 is the RNA-dependent RNA polymerase (RdRp), a key player in the viral replication and transcription processes and is a primary drug target in COVID-19 [58–61]. The first FDA-approved drug, Remdesivir, is the SARS-CoV-2 RdRp inhibitor sold under the name of Veklury and is administered by intravenous infusion in hospitalized COVID-19 patients [58–61].

Importantly, nsp3 and nsp5 are the two core viral proteases, the papain-like protease (PLpro) and the main protease, also called 3-chymotrypsin-like protease (3CLpro), respectively. These two viral proteases are responsible for cleavage of the viral polyprotein, pp1a or pp1ab [62–65]. 3CLpro (nsp5) undergoes autocleavage before liberating the majority of nsps (nsp4-16). Similarly, PLpro (nsp3) undergoes autocleavage, before the liberation of the remaining nsps (nsp1-4) [62–65]. 3CLpro is a conserved cysteine protease among coronaviruses. Since 3CLpro catalyzes the production of most nsps and plays a pivotal role in SARS-CoV-2 replication, it represents a highly attractive drug target for developing antivirals against COVID-19 [66–68].

This review provides an updated overview of the literature on the structure and function of 3CLpro, highlighting key findings that elucidate its role as the main protease of SARS-CoV-2. It discusses various high-resolution structural studies that have contributed to detailed atomic models of 3CLpro. Furthermore, the review underscores the significance of targeting 3CLpro in developing antiviral therapeutics against COVID-19. In this regard, a critical strategy involves identifying small molecules capable of binding to 3CLpro, focusing on both the active site and newly identified allosteric sites. The review delves into different types of inhibitors targeting SARS-CoV-2 3CLpro, including covalent inhibitors encompassing both peptide- and non-peptide-based small molecules. The non-covalent small molecules that bind the active site make competitive inhibitors; others that bind allosteric sites form non-competitive inhibitors With this knowledge, the extensive efforts in discovering inhibitor candidates against 3CLpro are also examined, noting that some have already been approved for clinical use in treating COVID-19 patients. Several 3CLpro inhibitors mentioned in this work are based on inhibitors developed against other related viruses such as SARS-CoV and MERS-CoV. These inhibitors show good activity against SARS CoV-2 and other related viruses. Newly developed SARS-CoV-2 protease inhibitors can potentially treat COVID-19 in addition to serving as broadspectrum inhibitors that could act on related coronaviruses, SARS-CoV-2 mutants and new pathogenic coronaviruses that may emerge in the future. The review presents the current state of research and provides insights into potential therapeutic avenues for combating COVID-19.

## Structural characterization of 3CLpro

Structural biology has made a remarkable contribution to our understanding of the replication mechanism of SARS-CoV-2. The structures of 13 SARS-CoV-2 proteins have been resolved, providing a wealth of data for deciphering the function of each protein and its potential use as a drug target against COVID-19 [69]. At the time of writing this review, a total of 698 structures for 3CLpro of SARS-CoV-2 have been deposited in the protein data bank since 2020. Of the total structures, 209 of which are apo-structures of 3CLpro. Most of the structures were resolved by X-ray crystallography, while four structures were resolved by cryo-EM. A wealth of knowledge was gained from the deposited structures, which advanced structure-aided drug design for screening of small molecule inhibitors against 3CLpro of SARS-CoV-2 [69]. Indeed, structural characterization is an invaluable tool for determining the inhibitory mechanisms of various drug candidates against 3CLpro. Approximately 320 resolved structures of 3CLpro in complex with inhibitors have been deposited in the protein data bank.

**Figure 2:**
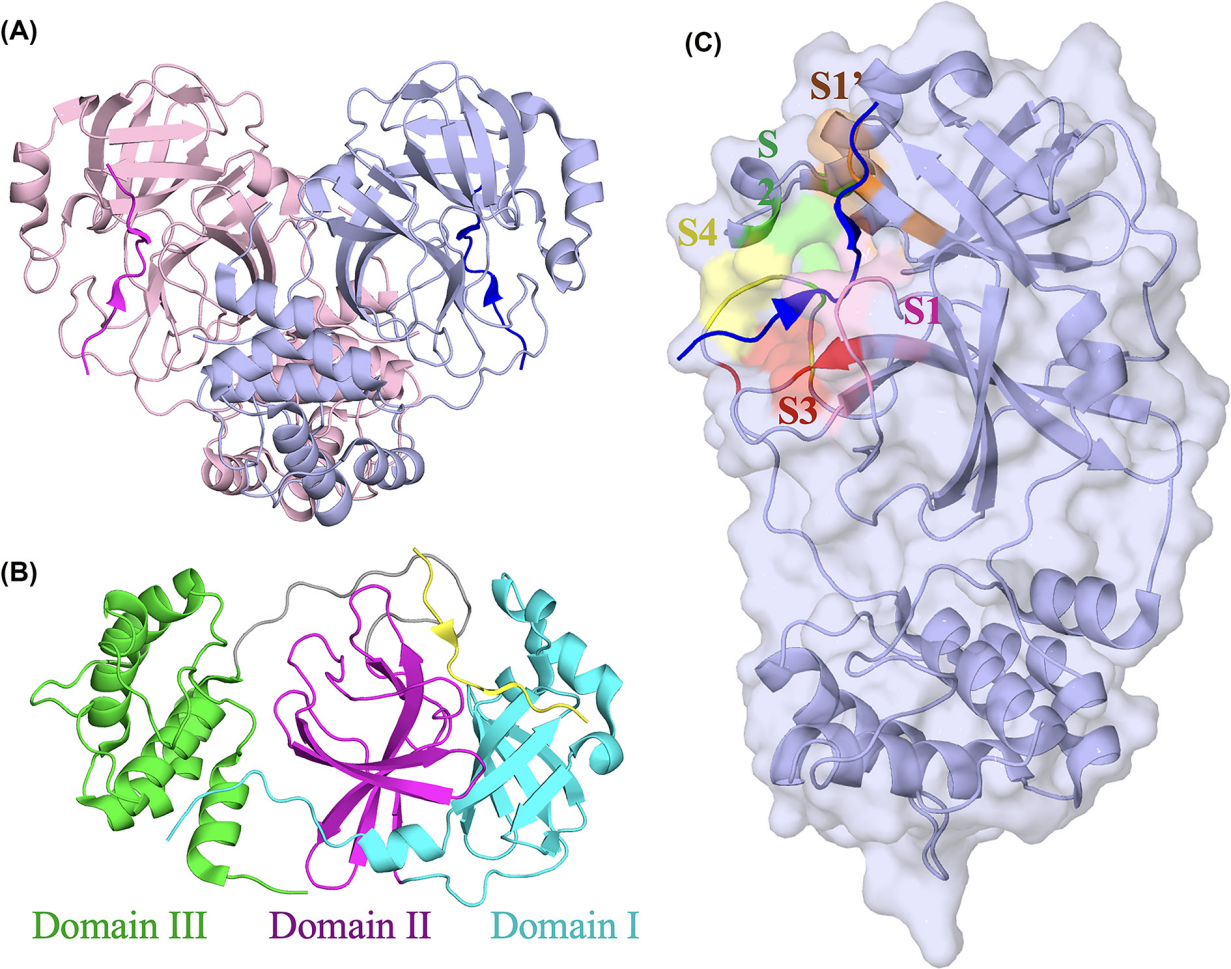
Structural Characterization of SARS-CoV-2 3CLpro (**A**) The active form of SARS-CoV-2 3CLpro is represented by the dimer with the two protomers colored pink and purple perpendicular to each other (PDB: 7T70). The structure is co-complexed with the peptide substrate. (**B**) SARS-CoV-2 3CLpro monomer is a 33.8 kDa protein with 306 amino acid residues. The figure represents one 3CLpro protomer in complex with the peptide substrate colored in yellow. 3CLpro consists of three domains: domain I (10-99) in cyan, II (100-182) in magenta, and domain III (201-203) in green. Domains I and II form a chymotrypsin-like fold, while domain III has an α-helical fold consisting of five α-helices. 3CLpro active site is located at the cleft between domains I and II. (**C**) The SARS-CoV-2 3CLpro active site pocket shows the subsites, S1′, S1, S2, S3, and S4, which are important for recognizing the polyprotein substrates.

3CLpro of SARS-CoV-2 is a 33.79 kDa protein consisting of 306 amino acid residues per monomer [70]. However, the homodimer is the catalytically active form of the protease, with the protomers arranged perpendicular to each other (Figure 2A). Each monomer of 3CLpro consists of three domains with domains I (10-99), II (100-182), and III (200-303), with the first two domains having a chymotrypsin-like fold consisting of six stranded β-sheets and a few α-helices (Figure 2B) [65]. The active site of 3CLpro is present at the cleft between domains I and II [70]. Domains II and III are connected by a long loop spanning residues 183-199, with domain III having an α-helical fold consisting of five α-helices (Figure 2B).

**Figure 3:**
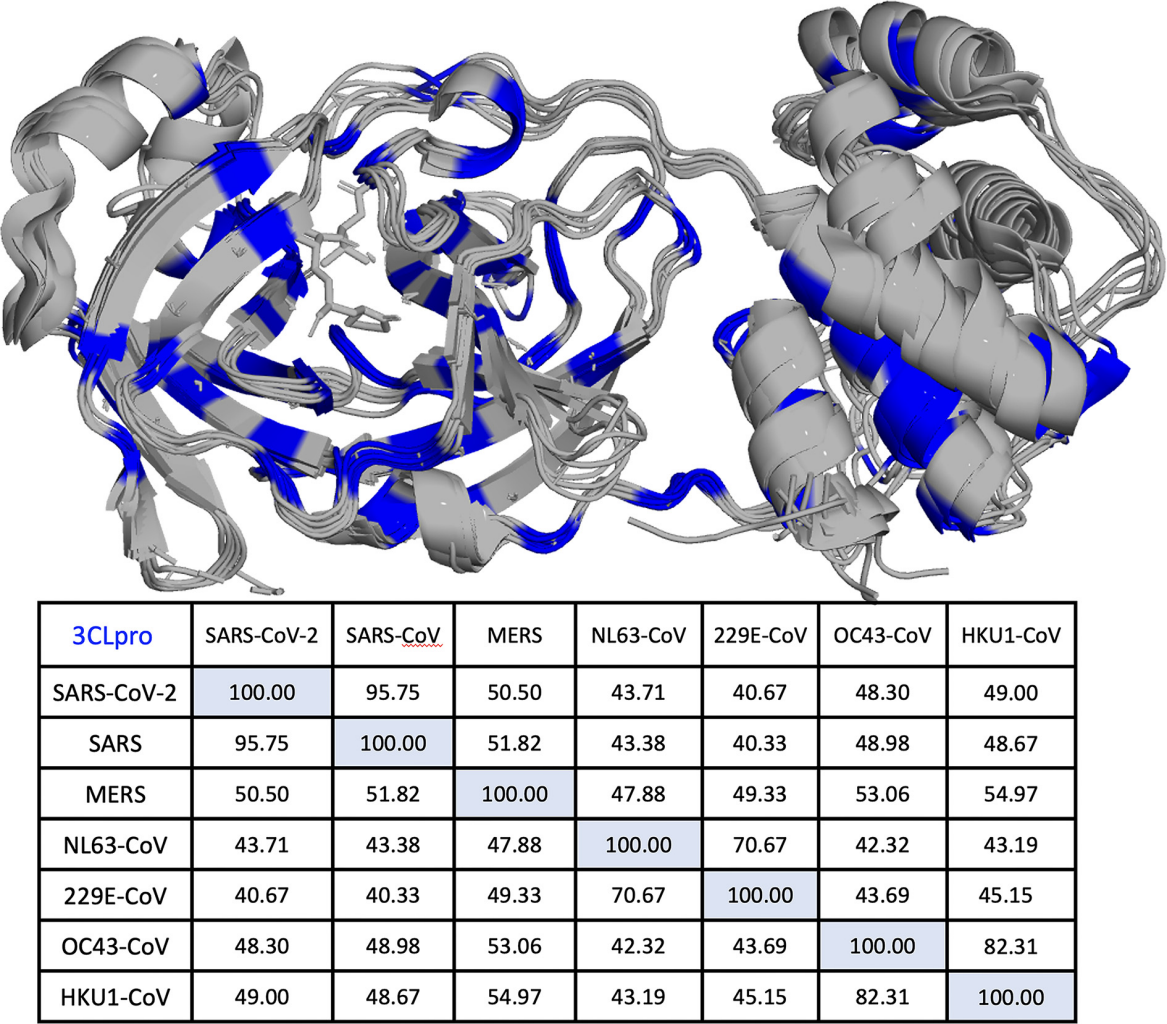
Sequence alignment and structural superposition of 3CLpro Structural superposition of 3CLpro from seven human coronaviruses, i.e. SARS-CoV-2, SARS-CoV, MERS-CoV, NL63-CoV, 229E-CoV, OC43-CoV, and HKU1-CoV, demonstrates highly conserved structural fold. Multiple sequence alignment of all 3CLpro sequences shows 84 fully conserved amino acids in 3CLpro enzymes of all human coronaviruses (colored in blue). It is observed that domain III is the most variable region, while the active site is the most conserved region. The conservation matrix of the seven different human coronaviruses reveals the highest identity score of 95.8% for 3CLpro sequences of SARS-CoV and SARS-CoV-2. In addition, a high identity score of 82.3% is observed between the 3CLpro sequences of OC43-CoV and HKU1-CoV, both β-coronaviruses. Similarly, a high identity score of 70.7% is observed between the 3CLpro of NL63-CoV and 229E-CoV, both α-coronaviruses.

Sequence alignment and structural superposition of 3CLpro from the seven human coronaviruses (i.e. SARS-CoV, MERS-CoV, SARS-CoV-2, NL63-CoV, 229E-CoV, OC43-CoV, and HKU1-CoV) reveal sequence similarity and conservation of key residues in this enzyme (Figure 3A). Multiple sequence alignment of all 3CLpro sequences shows 84 fully conserved residues, 50 with strongly similar physicochemical properties, and 24 with weakly similar properties. Of the conserved 3CLpro residues, domain III constitutes the most variable region, while the active site constitutes the most similar region. Indeed, the catalytic dyad residues, H41 and C145, are among the conserved residues in 3CLpro of all human coronaviruses. Additionally, the active site of 3CLpro consists of numerous fully conserved residues, N28, R40, Y54, S139, F140, S147, Y161, H163, E166, H172, D187, and Q192 that have been shown to be critical for maintaining 3CLpro function [71,72]. As for the allosteric sites of 3CLpro, the fully conserved residues N203, D289, E290, and Q299 are essential for maintaining the catalytic activity of 3CLpro [71,72].

Among the seven different human coronaviruses, the conservation matrix reveals the highest identity score of 96% for 3CLpro of SARS-CoV and SARS-CoV-2 (Figure 3B). A high identity score of 82% is observed between 3CLpro of OC43-CoV and HKU1-CoV, both β-coronaviruses. Similarly, an identity score of 71% is observed between 3CLpro of NL63-CoV and 229E-CoV, both α-coronaviruses. Structural and biochemical studies of 3CLpro identified two catalytic dyad residues, C145 and H41, that are important for catalysis [73]. A distance of 3.8 Å is recorded between Sγ on the side chain of C145 and Nε2 on the side chain of H41 of the catalytic dyad [74].

The crystal structures of 3CLpro in complex with the peptide substrate have been useful in characterizing the binding and cleavage sites of the viral polyprotein’s substrate [75,76]. Particularly, 3CLpro substrate-bound structures show notable conformational changes in the active site vicinity compared with the apo structure [75,76]. The dynamic and flexible nature of the 3CLpro active site is required to recognize a variety of substrates along the viral polyproteins [74]. The peptide substrate residues recognized by the 3CLpro active site are referred to, from N to C terminus, P4-P3-P2-P1-P1′-P2′-P3′ with the hydrolysis taking place at the scissile bond between P1 and P1′ [76,77]. Similarly, the 3CLpro active site is divided into multiple subsites, referred to as S1′, S1, S2, S3, and S4, that are important for recognizing of peptide substrates (Figure 2C). Naturally, 3CLpro recognizes diverse cleavage sequences but with a stringent requirement of glutamine at P1, which gets recognized by the S1 subsite in the active site of 3CLpro. S1 subsite lies next to the catalytic dyad residues, H41 and C145, and contains the residues, F140, L141, N142, G143, H163, E166, H172, and Ser1 of the other monomer [70,76–82]. S1 subsite residues form an intricate network of interactions with the peptide substrate to ensure glutamine specificity at P1 substrate position [70,76–82].

On the other hand, the S2 subsite features higher flexibility among the other subsites and preferentially binds Leucine or other hydrophobic residues (Phe/Val) of the substrate at P2 position [80]. S2 subsite cavity is formed by the side chain of amino acids H41, M49, Y54, and D187 [70,76,77,79–82]. S3 subsite is a solvent-exposed pocket that can accommodate several peptide substrates with diverse amino acid sequences, including positively charged (Lys/Arg), polar (Thr) or hydrophobic (Val/Met) amino acids. The S3 subsite is formed by the side chain of amino acids M165 and Q189 and the backbones of H41, E166, and Q189 [78,79,82]. The S4 subsite is a semi-enclosed hydrophobic pocket that can accommodate P4 of the peptide substrate with Ala or Val [75,76,78,82]. S4 is formed by the residues, M165, L167, F185, Q189, T190, and E192 [75,76,78,82]. Lastly, the S1′ subsite is a shallow pocket buried near the S1 subsite and is formed by the residues T24, T25, and L27 that can interact with smaller amino acids at position P1′ of the peptide substrates (Ser, Ala, or Gly) [75,76,82].

The subsites of 3CLpro reveal a sophisticated mechanism for substrate recognition and specificity, underlining the enzyme’s adaptability in cleaving the viral polyproteins. This emphasizes the unique features and flexibility of the subsites that are crucial for developing COVID-19 therapeutics.

## Functional characterization of 3CLpro

SARS-CoV-2 is a single-stranded positive-sense RNA virus whose viral genome has two large open reading frames (ORF1a/b) that get directly translated into two overlapping polyproteins, pp1a, and pp1ab, which encode the viral non-structural proteins (nsps) [83]. Nsps are individual viral subunits crucial for viral transcription, replication, and recombination [84,85]. The liberation of nsps along the polyproteins occurs through specific cleavage sites recognized by the two viral proteases, 3CLpro and PLpro [83,86]. The main protease of SARS-CoV-2, also known as 3-chymotrypsin-like protease, or 3C-like protease, (3CLpro), is a chymotrypsin-like cysteine protease. The name originates from its similarities with the main protease of picornavirus (3C proteinases), including substrate specificity and the requirement of a cysteine as the catalytic residue [65]. SARS-CoV-2 3CLpro is responsible for the proteolytic release of 11 nsps (including its own cleavage) from the polyproteins [83,87]. 3CLpro thus plays an indispensable role in the viral life cycle, without which coronaviruses cannot proliferate. 3LCpro recognizes and cleaves pp1a and pp1ab at specific amino acid sequences: Leu-Gln↓(Ser, Ala, Gly), where ↓ marks the site of cleavage [88–90].

### Catalytic mechanism of 3CLpro

3CLpro operates via a non-canonical Cys-His dyad, unlike the more commonly known Ser (Cys)-His-Asp(Glu) triad found in other serine or cysteine proteases [88–90]. The Cys-His catalytic dyad was also confirmed in SARS-CoV 3CLpro and MERS-CoV 3CLpro via crystal structure analyses [65,83,91]. In place of where a third member of a catalytic triad would be, the same position in the crystal structure of 3CLpro is occupied by a water molecule that is hydrogen-bonded to H41, H163, and D187 [74]. A biochemical study on 3CLpro also confirms the need for the C145-H41 dyad to carry out proteolysis in SARS-CoV-2 [92]. Alternative mutations at C145 or H41 to other amino acids with similar physicochemical properties failed to carry catalytic activity, suggesting the requirement for a Cys/His pair to carry out catalysis in 3CLpro of SARS-CoV-2 [92].

The chemical mechanism of SARS-CoV-2 3CLpro follows the typical reaction mechanism of other cysteine and serine proteases confirmed by pH studies with the peptide substrate cleavage carried out by the catalytic dyad H41-C145 upon binding the subsites of 3CLpro [73,93]. Catalysis is initiated by H41, which abstracts hydrogen from the thiol group of C145. Now a far better nucleophile, the resulting thiolate performs a nucleophilic attack on the carbonyl carbon of glutamine in the backbone of the substrate, forming a tetrahedral thiohemiacetal intermediate that contains an oxyanion group. This high-energy intermediate is stabilized by the hydrogen bonds formed between the main chain nitrogens’ of C145 and G143, and the oxyanion [94,95]. The collapse of the thiohemiacetal complex releases the C-terminal segment of the polypeptide substrate [81,96,97]. Finally, the thioester linkage between the substrate and 3CLpro is hydrolyzed by a water molecule, displacing the catalytic residue, C145, and releasing the N-terminal segment of the peptide substrate [81,96,97].

The enzymatic activity of 3CLpro follows a general base mechanism with a bell-shaped pH profile of 3CLpro was reported with the requirement for two amino acids with p*K*_a_ values of 6.9 ± 0.1 and 9.4 ± 0.1, corresponding to the ionizable side chains of the H41-C145 catalytic dyad residues, respectively [73,96,98]. The role of the conserved histidine residues H163, H164, and H172 in the active site of 3CLpro has been investigated, with only H163 playing a crucial role in maintaining 3CLpro activity [73]. The imidazole side chain of H163 has been shown to form a conserved hydrogen bond with the side chain of Gln (P1) of the peptide substrate [76].

### *In vitro* characterization of 3CLpro function

A major step in understanding the functional role of 3CLpro is to isolate it and study it *in vitro* [99–102]. 3CLpro purification, structural determination, and biochemical analyses have been extensively studied in the literature, with the untagged enzyme being more active compared with the tagged 3CLpro, which provides the basis for drug discovery [99–104]. The expression of 3CLpro in *Escherichia coli* and subsequent purification is typically followed by the FRET-based enzyme assay to characterize 3CLpro activity in the absence or presence of candidate inhibitors. Fluorescence resonance energy transfer (FRET) is a powerful tool widely used to probe all sorts of macromolecules, and the usage of FRET to study 3CLpro catalysis is no exception [105]. The current body of literature on 3CLpro often uses FRET-based assays to study the catalysis of 3CLpro, which involves the use of a short polypeptide, resembling the natural 3CLpro substrate, that is covalently modified to have a fluorophore on one end and a quencher on the other [99,101,106]. When the fluorogenic peptide is cleaved and the fluorophore and quencher pair separate, an increase in fluorescence is observed, allowing for the continued monitoring of 3CLpro activity [101]. A significant advantage of using FRET to probe catalysis is sensitivity, which is beneficial in screening large libraries of inhibitor candidates at low enzyme concentrations.

Another challenge in the drug discovery and design of small molecule inhibitors targeting 3CLpro for their use as COVID-19 antivirals is the use of dimethyl sulfoxide (DMSO) in the enzymatic assay of 3CLpro. DMSO is a highly polar aprotic solvent. It is frequently used to dissolve many hydrophobic small molecules with poor solubility. However, at higher concentrations, DMSO has the potential to denature proteins, making it challenging to have DMSO at concentrations high enough to solubilize small molecules without denaturing the protein target for drug screening. The DMSO denaturation effect is concentration-dependent, and specific proteins may be more resistant to denaturation than others. As a result, the DMSO effect has been investigated on the stability and activity of 3CLpro for its use in 3CLpro enzymatic assay [107]. Even though 20% DSMO reduced 3CLpro stability, it maintained 3CLpro in a catalytically active state. The melting point of 3CLpro was reduced from 55 °C to 45 °C in the absence and presence of 20% DMSO, respectively, with both melting points being above the temperature (30°C) used for *in vitro* enzymatic assay of 3CLpro. The presence of 20% DMSO enhanced the solubility of its peptide substrate, which enhanced the turnover rate of 3CLpro compared to lower DMSO concentrations in the assay [108].

### Naturally occurring mutants of SARS-CoV-2 3CLpro

Several new variants of SARS-CoV-2 have emerged since the start of the COVID-19 pandemic, such as the α-, β-, γ-, Δ-, Omicron, and Zeta variants [5]. This led to numerous mutations in 3CLpro that had different effects on its enzymatic activity and drug resistance. The circulating 3CLpro mutant from the SARS-CoV-2 sub-lineage (B.1.1.284), P108S, was found to reduce enzyme activity, reflecting milder clinical symptoms in patients with this point mutation [109]. Biochemically, the P108S mutant resulted in structural changes near the active site pocket and had a lower *k*_cat_/*K*_m_ value than WT enzyme [109]. These biochemical and structural changes introduced by the P108S mutant on 3CLpro may be the main contributor to the milder clinical course observed with the SARS-Cov-2 sub-lineage (B.1.1.284) carrying the P108S mutation. Comparatively, the Omicron SARS-CoV-2 (B.1.1.529) variant with the P132H point mutation in 3CLpro reduced the thermal stability of 3CLpro but did not affect its enzyme activity [110]. Clinically, the Omicron SARS-CoV-2 (B.1.1.529) variant is more transmissible with a notable ability to evade host immune responses than other SARS-CoV-2 variants [110].

Other globally circulating SARS-CoV-2 lineages (Lambda, Beta, Omicron, and Zeta), with 3CLpro mutations, G15S, T21I, L89F, K90R, P132H, and L205V, were found to have comparable enzyme activities without causing resistance to the approved 3CLpro drug, Nirmatrelvir [111]. Nevertheless, some mutations in the 3CLpro active site affected the inhibitor’s efficacy. An extensive analysis of 102 natural mutants of 3CLpro arising from different variants of SARS-CoV-2 including mutations within 3CLpro active site at S144(A/G/Y/M/F), M165T, E166 (A/G/V), H172 (Q/F), and eleven mutations at Q192 had notable resistance to the inhibitory effect of Nirmatrelvir despite showing comparable enzyme activity to 3CLpro WT [112]. In another study, active site mutations of 3CLpro, G143S, and Q189K were associated with low inhibitory activity of Nirmatrelvir, while M49I, G143S, and R188S mutants were associated with low inhibitory activity of Ensitrelvir, suggesting that different inhibitors may have different resistance profiles [113]. As a result, the continuous use of 3CLpro inhibitors may exert evolutionary pressure, leading to potential resistance. Hence, it is vital to understand the interplay between SARS-CoV-2 mutations, and potential 3CLpro inhibitor resistance [114]. One solution would be to adapt inhibitors to target evolving 3CLpro variants by using combinatorial approaches that employ different classes of inhibitors, such as allosteric inhibitors that bind at sites other than the active site, which would reduce the risk of resistance and enhance the overall efficacy of antiviral treatments against SARS-CoV-2.

## SARS-CoV-2 3CLpro as a drug target

Given its central role in the viral replication cycle, 3CLpro is an attractive target for the treatment of COVID-19. The identification of highly specific and potent inhibitors against 3CLpro of SARS-CoV-2 can stop or reduce the processing and production of new viral particles to enable the immune system to eliminate viral infection [46,115,116]. The screening of inhibitors against 3CLpro has been extensively studied, with a focus on repurposing existing approved drugs and developing new and novel antivirals against SARS-CoV-2 [117]. This can be achieved by targeting the protease active site or allosteric sites that have been identified to interfere with its proteolytic activity, thereby hindering viral replication [71,73,118]. The active site of 3CLpro has been used primarily for the identification and development of covalent and non-covalent competitive inhibitors [119]. These inhibitors could target the catalytic dyad H41 and C145, or the extended peptide binding pocket of 3CLpro [120].

### Covalent inhibitors of SARS-CoV-2 3CLpro

Overall, 3CLpro inhibitors can be classified into covalent and non-covalent inhibitors, with the former containing an electrophilic functional group called ‘warhead’, that forms a covalent bond with the catalytic residue C145 [121–123]. Covalent inhibitors are usually more potent and cannot be easily reversed or displaced by increasing the substrate concentration [116]. Covalent inhibitors are further classified as peptidomimetic or non-peptide small molecule inhibitors that form strong and specific covalent bonds with their target [124]. However, they could be toxic and have less favorable pharmacokinetic properties, necessitating the search for an alternative mode of inhibition, namely reversible non-covalent inhibitors [125]. On the other hand, non-covalent 3CLpro inhibitors, despite forming weak bonding interactions with either the active site or allosteric sites residues of 3CLpro, are less toxic and can make specific and effective inhibitors against 3CLpro [126]. For example, competitive inhibitors that bind non-covalently to the 3CLpro active site have an inhibitory effect that can be reversed in the presence of increasing substrate concentrations. However, allosteric inhibitors usually make non-competitive inhibitors that are unaffected by the presence of substrate as they do not share the same binding site. Various covalent and non-covalent 3CLpro inhibitors have been studied, and only those tested *in vitro* and/or *in vivo* are described here (Supplementary Tables S1–S3).

#### Peptide-based covalent 3CLpro inhibitors

**Figure 4:**
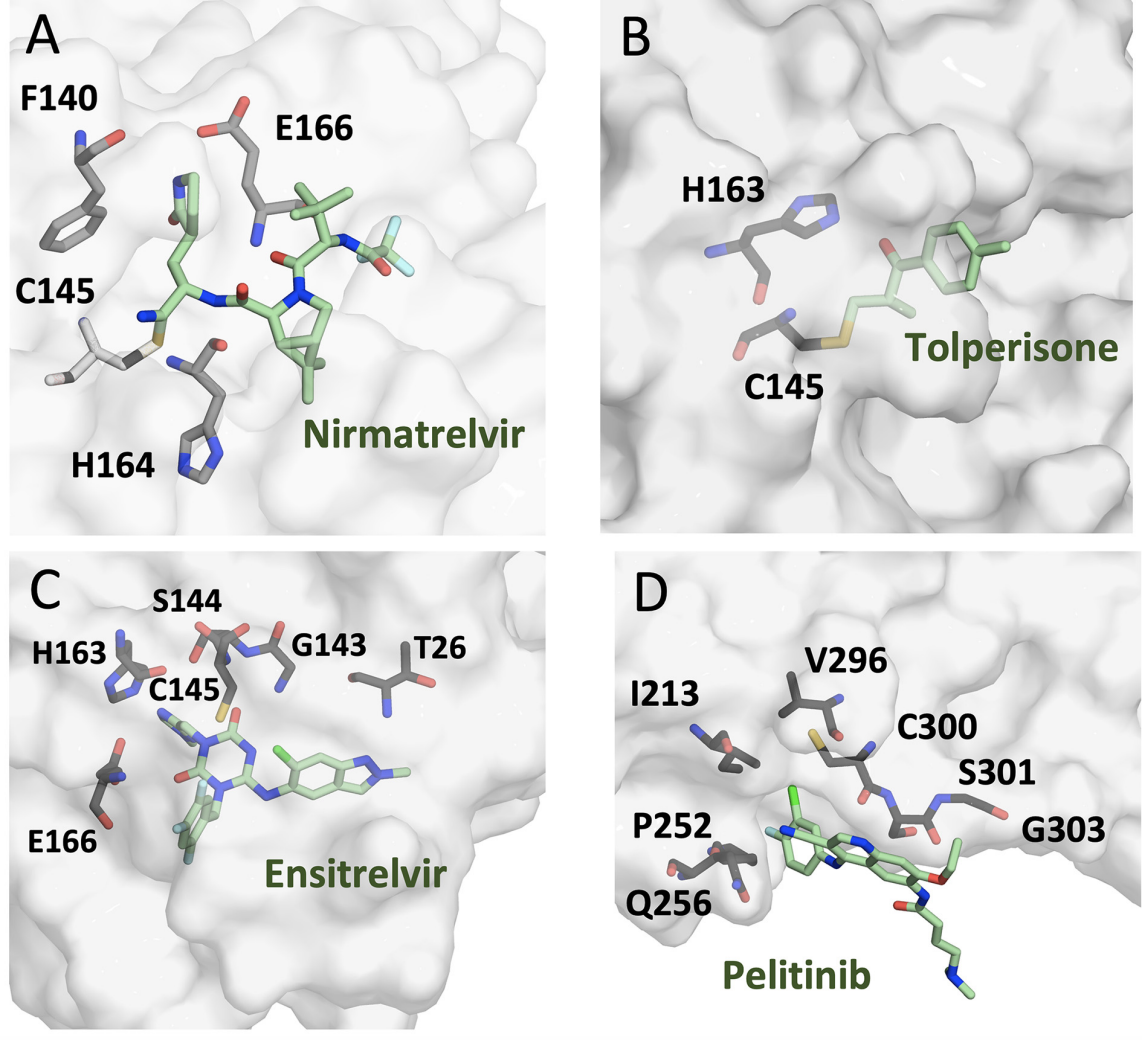
Structural representation of SARS-CoV-2 3CLpro in complex with several specific inhibitors to elucidate various inhibition mechanisms (**A**) Structural representation of SARS-CoV-2 3CLpro in complex with Nirmatrelvir (PDB: 7SI9) demonstrating the binding mode of the peptide-based inhibitor (Nirmatrelvir) that forms a covalent bond with the catalytic residue, Cys145, of 3CLpro via its nitrile warhead. Nirmatrelvir is the first oral antiviral drug developed by Pfizer under the name of Paxlovid, which was approved by the FDA for treating COVID-19 patients. (**B**) Structural representation of SARS-CoV-2 3CLpro in complex with the non-peptide-based covalent inhibitor, Tolperisone (PDB: 7ADW), demonstrating the binding mode of Tolperisone that forms a covalent bond with the catalytic residue, Cys145, of 3CLpro. (**C**) Structural representation of SARS-CoV-2 3CLpro in complex with the competitive non-covalent inhibitor, Ensitrelvir (PDB: 8DZ0), demonstrating the binding mode of Ensitrelvir that forms several non-covalent bonding interactions with key active site residues of 3CLpro. (**D**) Structural representation of SARS-CoV-2 3CLpro in complex with the non-competitive allosteric inhibitor, Pelitinib (PDB: 7AXM), demonstrating the binding mode of Pelitinib that forms several non-covalent bonding interactions with residues in a hydrophobic pocket formed at the interface of domains II and III of SARS-CoV-2 3CLpro. The crystal structure of 3CLpro in complex with Pelitinib reveals the inhibition mechanism of pelitinib that interacts with S301 in domain III and pushes against the active site residue, N142, leading to the destabilization of the catalytic S1 subsite that is important for the proteolytic activity of 3CLpro.

Peptide-based covalent inhibitors, referred to as peptidomimetics, are small molecules that mimic the structure of 3CLpro’s natural polyprotein substrates, yielding stable inhibitors with improved pharmacokinetic profiles (Supplementary Table S1) [127]. Several potent peptidomimetic inhibitors have now proceeded into clinical trials or were granted FDA approval for treating COVID-19 patients. Firstly, Nirmatrelvir (PF-07321332) is the first oral antiviral drug developed by Pfizer, under the name of Paxlovid, and approved by the FDA for treating COVID-19 patients [128,129]. Nirmatrelvir is a peptidomimetic inhibitor of SARS-CoV-2 3CLpro that forms a reversible covalent bond with the side chain of C145 via its nitrile warhead (Figure 4A). It was further developed by systemically improving the pharmaceutical properties of compound PF-00835231 initially designed for SARS-CoV 3CLpro [130,131]. Nirmatrelvir plus ritonavir was approved by FDA for emergency treatment of mild-to-moderate and high-risk COVID-19 cases and was shown to be effective against the emerged SARS-CoV-2 variants [132,133].

Ritonavir (NDA# 020945) is an FDA-approved drug used in combination with the protease inhibitor, lopinavir, to treat HIV infection [134]. However, a test of the combined treatment of ritonavir with lopinavir showed no effective response in hospitalized COVID-19 cases [135]. A closely related analog to nirmatrelvir is Lufotrelvir (PF-07304814), developed as a phosphate prodrug of the competitive inhibitor PF-00835231 [136]. As a phosphate prodrug, Lufotrelvir is metabolized *in vivo* by the alkaline phosphatases to release the active metabolite, PF-00835231, that can covalently bind and inhibit 3CLpro [137–140]. In preclinical studies, compound PF-00835231 was associated with low solubility (<1 mg/ml), bringing the need to develop a prodrug, Lufotrelvir, which has been reported to exhibit improved aqueous solubility (>200 mg/ml) needed for drug delivery [137–140].

Efforts to develop novel peptidomimetic competitive inhibitors against SARS-CoV-2 3CLpro led to the design of the reversible broad-spectrum covalent inhibitors, GC376 and GC373, where the former is the dipeptidyl aldehyde bisulfite prodrug of the latter. Biochemical, NMR, and cell assays confirmed their covalent binding via the aldehyde bisulfite warhead with C145 at the active site of SARS-CoV-2 3CLpro [141–146]. The synthesis of a deuterated variant of GC376 yielded compound 2 that demonstrated potent inhibition against 3CLpro, with the crystal structure of 3CLpro in complex with compound 2 revealing covalent binding with C145 of 3CLpro [147]. The introduction of deuterium into GC376 resulted in improved pharmacokinetics and reduced toxicity, as shown in cell-based and mouse models. A series of dipeptide and tripeptide inhibitors, analogs of GC376 (compounds MPI5 and MPI8), were synthesized and tested against 3CLpro, with crystallographic studies confirming the formation of reversible covalent bonds with C145 of 3CLpro [148]. In addition, live viral cell-based experiments showed that MPI5 and MPI8 effectively prevent the cytopathogenic effect induced by SARS-CoV-2 infection [148].

A computational analysis of the substrate binding pocket of SARS-CoV-2 3CLpro led to the structure-based drug design of novel peptidomimetic inhibitors with aldehyde warheads, compounds 11a and 11b, both of which exhibit covalent inhibition as confirmed by crystallography and FRET-based assays [77]. Another study reported the design of potent peptide-based inhibitors with aldehyde warheads, compounds 2(a-o) and 3(a-o), that were synthesized as deuterated and non-deuterated dipeptidyl aldehydes with a cyclohexane moiety [149]. These compounds showed effective inhibition against SARS-CoV-2 3CLpro in both biochemical and cell-based assays. In addition, the inhibitors were found to be potent against MERS-CoV 3CLpro. The high-resolution cocrystal structure of inhibitor compound 2a elucidated the mechanism of action by forming a covalent bond to C145 at the active site of SARS-CoV-2 3CLpro [149].

Another structural class of peptidomimetic inhibitors is those that possess an α-ketoamide warhead that forms a covalent interaction with C145 of 3CLpro. Among the first novel candidates is compound 13b, which has been shown to be a broad-spectrum inhibitor against 3CLpro of α- and β-coronaviruses and the main protease of enteroviruses [81,150]. AT1001 is a peptidomimetic inhibitor structurally similar to compound 13b and has been reported as a potential inhibitor that binds 3CLpro active site [151]. Alternative novel tripeptide derivatives of AT1001 were designed with improved inhibition of the 3CLpro activity and SARS-CoV-2 viral replication [152]. The derivatives were found to have a tighter affinity with 3CLpro from X-ray crystallography studies and enhanced antiviral activity from *in vivo* inhibition experiments [152].

In light of drug repurposing, several clinically approved drugs were screened as peptidomimetic inhibitors of 3CLpro, taking into account their existing clinical data, such as duration of action, dose, and their cytotoxic and side effect levels. For example, boceprevir (Victrelis, NDA #202258), an FDA-approved drug against the main protease of the hepatitis C virus, was tested for inhibition of SARS-CoV-2 3CLpro activity [153,154]. Boceprevir, a synthetic α-ketoamide tripeptide, was shown to reversibly bind the active site of 3CLpro in both biochemical and live viral replication assays [146,155–157]. Further derivatives of boceprevir were synthesized via a structure-based design approach and extensively characterized against 3CLpro [158–160]. Boceprevir analogs demonstrated higher antiviral efficacy in cellular assays for specifically binding and inhibiting SARS-CoV-2 3CLpro. The crystal structure of 3CLpro in complex with boceprevir was used in a structure-guided study to design a newly approved oral drug against SARS-CoV-2 3CLpro. The new drug, Simnotrelvir (SIM0417), marketed under the name of XIANNUOXIN™, is used in combination with ritonavir to treat COVID-19 patients with mild-to-moderate symptoms [161,162]. In another recent study, the structure of boceprevir was used as a scaffold to design compound ML2006a4; an orally bioavailable peptide-based inhibitor of SARS-CoV-2 3CLpro with improved bioavailability, increased affinity, and reduced sensitivity to mutations as compared to nirmatrelvir [163].

Another potent α-ketoamide inhibitor of SARS-CoV-2 3CLpro is the compound RAY1216, referred to as leritrelvir, which has proceeded into phase 3 clinical trials against COVID-19 [164,165]. Leritrelvir was reported to exhibit longer drug-target residence time, 104 min compared with 9 min for Nirmatrelvir, indicating a slower dissociation of leritrelvir from the enzyme-inhibitor complex as compared with Nirmatrelvir. Structural analysis reveals the covalent reversible interaction of leritrelvir α-ketoamide warhead with C145 of 3CLpro [164,165].

Similar to boceprevir, other clinical antiviral drugs that target the main protease of hepatitis C virus, such as telaprevir, narlaprevir, paritaprevir, and tipranavir, were found to covalently bind and inhibit SARS-CoV-2 3CLpro [157,166,167]. Importantly, boceprevir and tipranavir were used as structural templates to design derivatives, MI-09, MI-30, with improved antiviral activity against 3CLpro [168,169].

Covalent peptide-based inhibitors of 3CLpro with various reactive electrophiles were tested, leading to the discovery of potent inhibitors with high target specificity, Jun9-62-2R and Jun9-88-6R. These inhibitors possess dichloroacetamide and tribromoacetamide warheads, respectively, which covalently bind to C145 of SARS-CoV-2 3CLpro. Importantly, unlike GC-376, compounds Jun9-62-2R and Jun9-88-6R were found to be specific inhibitors against 3CLpro of SARS-CoV-2 without inhibiting host proteases such as Cathepsin L [170]. Following the same design rationale, several reactive warheads were tested, leading to the discovery of further highly potent covalent 3CLpro inhibitors, namely Jun10541R and Jun10963R with nitrile and dually activated nitrile warheads. These inhibitors showed promising antiviral activity and enzymatic inhibition, with IC_50_ of 0.50 μM and 0.56 μM, respectively, against SARS-CoV-2 [171].

The peptidomimetic inhibitor, CDI-45205 (undisclosed structure), is selected by Cocrystal Pharma and has advanced to late preclinical testing stages, showing inhibitory activity against 3CLpro from MERS-CoV and SARS-CoV-2 [63,172]. Other potential peptidomimetic 3CLpro inhibitors derived from drug repurposing are AG7404 and AG7088 (Rupintrivir). Both are orally available inhibitors originally developed to combat human enteroviruses (the causative agent of the common cold) and have progressed to phase II/III clinical trials [173,174]. Both compounds inhibit 3CLpro of SARS-CoV and SARS-CoV-2 in biochemical and antiviral cell-based assays by direct binding to the active site, with AG7404 showing higher inhibitory potency than AG7088 [175].

Previously reported non-covalent inhibitors of SARS-CoV 3CLpro, such as compound X77 containing an imidazole ring, were used to develop potent covalent inhibitors of SARS-CoV-2 3CLpro. Specifically, compounds 14a and 16a were designed by replacing the imidazole ring of the non-covalent inhibitor X77 with several reactive warheads, resulting in improved inhibitory activity of the new covalent inhibitors [176]. Overall, peptide-based covalent competitive inhibitors of 3CLpro show promising outcomes for their roles as antivirals. Their ability to specifically bind and interact with C145 of the catalytic dyad has proven their ability to specifically inhibit the catalytic activity of SARS-CoV-2 3CLpro.

#### Non-peptide-based covalent 3CLpro inhibitors

Non-peptide-based small molecules covalently binding the 3CLpro active site were discovered by computational and high-throughput screening of existing approved drugs, natural products, or synthetic chemicals (Supplementary Table S1) [177,178]. For example, Tolperisone, a muscle relaxant, is an investigational drug for use in neurologic disorders [179]. Tolperisone, a β-aminoketone, is regarded as the parent compound or prodrug that gets decomposed into a Michael acceptor breakdown product [117,179]. In turn, tolperisone binds covalently to C145 in the active site of SARS-CoV-2 3CLpro. X-ray crystallography and cell-based experiments have shown that the breakdown product of Tolperisone, i.e. the ketone element, binds covalently to the 3CLpro active site (Figure 4B) [179,180]. In addition to its covalent binding interactions, the aromatic ring of the breakdown product of Tolperisone occupies the S1 subsite and forms van der Waals interactions with the backbone atoms of F140, L141, and E166 [179].

Ebselen is another covalent inhibitor that is an investigational compound originally developed as an antioxidant mimic of glutathione peroxidase [181,182]. Several studies reported that Ebselen and its structural derivatives act as competitive inhibitors of 3CLpro. This is demonstrated by co-crystal structures in which Ebselen forms a covalent bond with C145 and is further stabilized by hydrogen bonding interactions with other active site residues [130,146,166,183–186]. Also, Carmofur, an antineoplastic drug employed in treating certain types of cancer, is reported to covalently bind the active site of SARS-CoV-2 3CLpro [184]. Carmofur contains an electrophilic carbonyl reactive group that targets the catalytic cysteine of 3CLpro [184].

Using structure-based drug design, Carmofur derivatives were developed to improve their binding at the S2 and S4 subsites of SARS-CoV-2 3CLpro, leading to more potent indole-based derivatives with a reduction in the half-maximal inhibitory concentration (IC50) values from 5 to 0.4 µM for Carmofur and its derivatives, respectively [187]. However, Carmofur did not exhibit cellular antiviral activity up to 100 μM in various cell-based enzyme assays, likely due to loss of inhibitory activity on 3CLpro under reductive conditions.

Disulfiram is an FDA-approved (NDA # 88-482) medication for alcoholism that primarily inhibits aldehyde dehydrogenase activity [188]. Although disulfiram was shown to covalently bind the active site of 3CLpro [70], it did not show an effective reduction in cellular antiviral activity [189]. Nevertheless, clinical studies reported the ability of disulfiram to reduce the severity of COVID-19 symptoms [190,191]. Although Ebselen, Carmofur and Disulfiram were reported as competitive covalent inhibitors of 3CLpro and other cysteine proteases. The inhibitory effect of these compounds was shown to be abolished upon adding the reducing agent, dithiothreitol (DTT), indicating that they do not form a specific thiol interaction with the catalytic active site cysteine of the cysteine protease [189].

### Non-covalent inhibitors of SARS-CoV-2 3CLpro

The choice between covalent and non-covalent inhibition depends on the characteristics of the target enzyme, the desired duration of action, and the need for selectivity [192]. Both inhibitors have been successfully employed in various therapeutic applications, and the decision often involves balancing the efficiency and efficacy of the small molecule target [125]. Reversible binding interactions of the non-covalent inhibitors provide regulatory control over the inhibition duration and could overcome the potential toxic side effects of covalent inhibitors [125,193,194]. They do possess less potential for toxicity compared with covalent inhibitors that form irreversible and potentially harmful interactions [195]. Non-covalent 3CLpro inhibitors can be divided into competitive and non-competitive inhibitors that bind the active site or allosteric sites, respectively (Supplementary Tables S2 and S3).

#### Competitive 3CLpro inhibitors

Ensitrelvir (or S-217622), marketed as Xocova, is the first clinically approved non-peptide competitive inhibitor that targets the active site of 3CLpro through non-covalent interactions [196,197]. Ensitrelvir forms several non-covalent bonding interactions with key active residues of 3CLpro (Figure 4C). Ensitrelvir is an orally administered drug that was granted emergency use authorization (EUA) in the US and Japan following its success in treating COVID-19 cases in phase 2/3 clinical trials [198]. Another non-covalent competitive inhibitor of 3CLpro is Perampanel, a glutamate receptor antagonist and an FDA-approved drug for the treatment of seizures. Perampanel and its structural analogues have been co-crystallised with 3CLpro, demonstrating non-covalent bonding interactions with the active site of 3CLpro [199]. Similarly, ML188 and ML300 are two non-covalent competitive inhibitors originally discovered from a high-throughput screening hit against 3CLpro of SARS-CoV in 2013 and have been shown to be effective in inhibiting 3CLpro of SARS-CoV-2 [200–202]. Based on the structure of the noncovalent inhibitor ML300, a structure-based design of several ML300 analogs led to the discovery of compound CCF0058981 as a non-covalent competitive inhibitor of SARS-CoV-2 3CLpro which exhibited a nanomolar inhibition of 3CLpro enzymatic activity as well as effective antiviral activity in cell-based models [200].

Using structure-based drug design and the one-pot Ugi four-component reaction, several ML188 analogs were synthesized and tested, resulting in the discovery of a selective and potent compound, 23R. It is a non-covalent 3CLpro inhibitor that binds between S2 and S4 subsites. Compound 23R showed a low IC_50_ value of 0.20 μM, demonstrating a 54-fold increase in inhibitory effect against 3CLpro compared with the parent compound ML188 [203]. Another novel non-covalent inhibitor of SARS-CoV-2 3CLpro is the compound MCULE-5948770040, which has been identified through high-throughput virtual screening. Biochemical and structural analyses demonstrate that MCULE-5948770040 has a low IC_50_ value. It binds noncovalently to the active site and occupies subsites S1 and S2 of 3CLpro [204].

Another non-covalent competitive inhibitor of 3CLpro is masitinib, a tyrosine kinase inhibitor, which was reported to decrease SARS-CoV-2 viral production [205]. In addition, Mastinib notably decreased SARS-CoV-2 viral load in mice and reduced inflammatory cytokines in the lungs [205]. Importantly, Masitinib is an FDA-approved drug in phase 3 clinical trial (NCT05441488), and a candidate for treating progressive forms of multiple sclerosis [206]. Its clinical use, in combination with Isoquercetin, has proven to be effective for the early treatment of COVID-19 (NCT04622865). Additionally, Baricitinib, an FDA approved oral tyrosine kinase inhibitor sold under the name Olumiant, is a competitive small molecule inhibitor of SARS-CoV-2 3CLpro with an IC_50_ in the micromolar range [207,208]. Baricitinib was granted an FDA EUA for hospitalized COVID-19 patients [209].

Another class of non-covalent competitive 3CLpro inhibitors is derived from natural compounds that treat viral infections and boost the host immune response [210–216]. These products have shown remarkable therapeutic benefits in previous coronavirus outbreaks such as SARS-CoV and MERS-CoV. Several natural antioxidant and anti-inflammatory polyphenols were found to be competitive inhibitors of SARS-CoV-2 3CLpro, showing robust antiviral effects in cell-based and biochemical assays [210–216]. These compounds are naturally found in various fruits, vegetables, leaves, and grains. They hold promise for various conditions, including viral infections, immune disorders, and cancer [217–221]. Some of these polyphenols include quercetin, resveratrol, rutin, ellagic acid, curcumin and EGCG (Supplementary Table S2). In fact, quercetin is among the most potent competitive inhibitors (IC50 of 7.40 µM) of 3CLpro and is under clinical trial (NCT04861298) for early-stage and mild-to-moderate symptomatic COVID-19 outpatients [222–224]. In addition, quercetin received the FDA GRAS (Generally Recognized As Safe) status for use as a dietary supplement [223,224]. Resveratrol, a polyphenol inhibiting 3CLpro activity *in vitro*, also inhibited SARS-CoV-2 in cell culture assays [214,225,226]. Resveratrol was in a clinical trial (NCT04400890) and demonstrated a lower incidence of COVID-19 hospitalization, ER visits, and pneumonia [227].

Recent dose–response studies aiming to target 3CLpro activity revealed the binding of curcumin to the 3CLpro active site with low micromolar IC_50_ values [214,228]. The polyphenolic epigallocatechin gallate, otherwise known as EGCG, was also reported to act as a broad-spectrum antiviral due to its effective inhibition of viral activity in adenovirus, influenza virus, zika virus, herpesvirus, hepatitis virus, and COVID-19 [211,229]. EGCG demonstrated an IC_50_ of 7.5 µM, similar to quercetin, and had no cytotoxicity on cultured cells [230].

#### Allosteric 3CLpro inhibitors

For most computational drug screening studies, the identification of allosteric inhibitors can be challenging, as the active site is usually the main target for screening of candidate hits that lead to the identification of competitive inhibitors. On the other hand, allosteric sites are surface pockets on a protein target that are distant from the active site and are capable of regulating its function [231]. As a result, inhibitors that bind the allosteric site may induce a conformational change or disrupt bonding interactions, thereby inhibiting the enzyme’s catalytic function. For this purpose, allosteric inhibitors would make potent inhibitors with increased binding affinity, inhibition efficacy, and target specificity [231]. Research efforts in this direction are essential for expanding our understanding of the structural and functional aspects of 3CLpro and identifying novel therapeutic intervention strategies.

The close relationship between dimerization and catalysis in 3CLpro is well documented and can lead to identification of allosteric sites for the virtual screening and identification of non-competitive inhibitors. Many reports have shown that the protease is unconditionally required to be a dimer to stay active [100,232–234]. For example, the R298A mutant was shown to disrupt the 3CLpro dimerization, which yielded an inactive 3CLpro SARS-CoV-2 [232]. Comparing the crystal structures of 3CLpro WT (dimer) and R298A (monomer) reveals one structural distinction, where a short stretch of residues (Gly138 to Leu141) in the dimeric state forms a loop, while the same stretch of residues in the monomeric state assembles into a 3_10_-helix [235]. This slight difference leads to local changes in the structural fold and elimination of key amino acid interactions, particularly at the loop formed by G143-S144-C145. These structural changes lead to removing the ring-stacking interaction between F140 and H163. These small perturbations in the fold lead to the collapse of the substrate-binding pocket and oxyanion hole, rendering the protease functionally inert. Another study investigated the role of nine amino acid residues in three key dimer interface sites, including the following paired residues: S1/E166, S10/K12/E14, and R4/E290/Q299/S139 [233]. Alanine mutants at sites 2 and 3 (i.e. at S10, E14, R4, E290, and Q299) eliminated enzyme activity. Nevertheless, some catalytically inactive mutants (R4A, R4Q, S10A/C, E14A/D/Q/S, E290A, and Q299A/E) were present as dimers, suggesting that dimerization alone is not indicative of catalytically active 3CLpro [233]. Moreover, the region around residues E288, D289, and E290 is near the N-finger residues involved in dimerization [232,236]. These key sites that control the activity of 3CLpro can be used as allosteric sites for the virtual screening and identification of non-competitive inhibitors.

Other potential allosteric sites of SARS-CoV-2 3CLpro arise at the interface of domains II and III, forming pockets that can serve as allosteric sites [71,179,237–239]. Key residues at these sites were found to modulate the activity of 3CLpro. For example, N203 is part of a druggable site in the groove between domains II and III, forming polar interactions with the backbone carbonyl oxygens of G109 and D289 [71,236,239–241]. The bonding interactions facilitated by N203 are important for the catalytic activity and the thermodynamic stability of 3CLpro, whereby the mutants N203A, N203Q and N203D were all catalytically inactive [71]. Another allosteric pocket contains D295, R298, and Q299, forming a network of interactions required to connect domains II and III [71,233,242]. In addition, the interdomain interactions between the allosteric residues R131, N133, D197, and D289 are crucial for the activity and stability of 3CLpro as they maintain proper orientation and interactions between domains II and III as well as the loop spanning residues 183 to 199 [71,72]. The newly identified allosteric sites of 3CLpro can be utilized for the virtual screening and identification of small molecules that may function as antivirals against COVID-19.

While extensive studies reported competitive 3CLpro inhibitors, fewer studies reported allosteric 3CLpro inhibitors that can bind to sites distant from the active site thus inhibiting 3CLpro without directly competing with the peptide substrate (Supplementary Table S3). A limited number of studies on allosteric 3CLpro inhibitors are reported in the literature, including biochemical or X-ray crystallography experiments. A hydrophobic pocket formed at the interface of domains II and III of SARS-CoV-2 3CLpro was shown to form a complex with three small molecule inhibitors, pelitinib, ifenprodil and RS-102895, with the first exhibiting the highest inhibitory activity [179]. Pelitinib is an investigational anticancer drug developed as an epidermal growth factor receptor (EGFR) inhibitor [243]. A crystallographic study showed that pelitinib binds to an allosteric pocket enclosed by residues I213, L253, Q256, V297, and C300 (Figure 4D). The binding site and inhibition mechanism of pelitinib were revealed by the co-crystal structure with 3CLpro, showing interactions with S301 in domain III and pushing against the active site residue, N142, leading to the destabilization of the catalytic S1 subsite that is important for the proteolytic activity of 3CLpro [179].

A co-crystal structure of AT7519 with SARS-CoV-2 3CLpro identified its binding site at the dimer interface involving domains I and II [179,244]. AT7519 is an inhibitor of cyclin-dependent kinases and is currently being investigated as an anticancer drug [179]. The crystallographic data showed that the chlorinated benzene ring of AT7519 forms multiple van der Waals and polar interactions with residues at the dimer interface pocket that are in loop 107-110, D153, V202, and T292. Importantly, AT7519 forms a salt bridge with R298, which plays a critical role in the dimerization of 3CLpro and the maintenance of the S1 subsite [179]. The ability of AT7519 to bind to R298 and interfere with its ability to dimerize the protease is an effective strategy to inhibit the catalytic function of 3CLpro. Another non-competitive inhibitor of 3CLpro is Apixaban, an FDA-approved oral anticoagulant for treating thromboembolic disease [245]. A biochemical study showed Apixaban to inhibit 3CLpro by binding to an allosteric site via a non-competitive inhibition mechanism [246]. Using cell-based screening, compound 172 was reported to inhibit 3CLpro of several SARS-CoV-2 variants of concern. Docking analysis revealed that compound 172 could bind to an allosteric site at the dimer interface formed by the residues: Met6, Phe8, Tyr 118-Ser123, Leu 141, Ile152, Asp153, Phe 294, Arg298, Asn299, and Ser 301. Importantly, compound 172 showed drug synergy with nirmatrelvir in biochemical assays as well as *in vivo* antiviral activities in Hamsters and mice models [247]. In addition, a potential non-competitive inhibitor of 3CLpro is Agathisflavone, a bioflavonoid with wide-ranging biological activities. A recent biochemical study suggests that the inhibition pattern of Agathisflavone against 3CLpro is non-competitive with low micromolecular EC_50_ values (4.32 µM) [248].

## Conclusions and future perspectives

This review discusses the structural and functional aspects of 3CLpro and highlights its role as the main protease of SARS-CoV-2. In particular, this review serves as a resource to recognize the progress in identifying and developing 3CLpro inhibitors to be used as antivirals against COVID-19, some of which are already approved for clinical use in COVID-19 patients. Several 3CLpro inhibitors mentioned here have been developed against other human viruses such as SARS-CoV and MERS-CoV. These inhibitors show good activity against SARS CoV-2 and other related viruses. This is not surprising considering the high structural similarity and conserved active sites of the 3CLpro proteases in coronaviruses and other human viruses, as shown in Figure 3. The newly developed SARS-CoV-2 protease inhibitors have the potential not only to treat COVID-19 but also to act as broad-spectrum inhibitors. They could be effective against related coronaviruses, SARS-CoV-2 variants, and new pathogenic coronaviruses that may emerge in the future.

The emerging SARS-CoV-2 variants exhibit a substantial mutation rate in various viral proteins, including 3CLpro. These mutations can lead to potential resistance and reduction of inhibition potency in the developed 3CLpro inhibitors, necessitating the ongoing development of antiviral inhibitors [72]. Monitoring viral sequences is crucial for detecting mutations and their impact on public health. Adapting inhibitors to target evolving 3CLpro variants is essential, with combinatorial inhibitor therapies to address specific mutations. Combining inhibitors targeting different binding sites could reduce the risk of resistance and enhance the overall efficacy of antiviral treatments. Our current understanding of 3CLpro of SARS-CoV-2 underscores the need to integrate novel computational approaches, such as artificial intelligence (AI) and machine learning (ML), in the drug discovery for 3CLpro inhibitors to combat COVID-19.

In addition, this review emphasizes the importance of exploring the surface pockets of 3CLpro as potential allosteric sites. This approach can expand inhibitor design beyond the active site with computational methods utilizing the allosteric sites for virtual small molecule screening in the identification of antivirals. Targeting diverse surface pockets enhances drug design, providing options for allosteric modulation, reducing resistance risk, and ensuring the high efficacy of 3CLpro inhibitors.

Virtual screening and ligand design processes have greatly benefited from computational methods to evaluate large compound libraries, predicting the binding affinity to 3CLpro. In fact, integrating AI and ML enhances accuracy by learning from high-quality experimental data, predicting ligand binding, and optimizing molecular structures for improved interactions [249]. Following the virtual screening, drug design processes would utilize molecular dynamics simulations and quantum mechanical calculations to fully comprehend ligand-protein interactions, where AI and ML would aid in predicting molecular properties, optimizing drug candidates, and identifying potential side effects to improve the drug design process. This review highlights the importance of exploring surface pockets of 3CLpro to expand inhibitor design beyond the active site and utilize computational methods for virtual small molecule screening to identify drug candidates. Targeting diverse surface pockets enhances drug design, providing options for allosteric modulation, reducing resistance risk, and ensuring the selectivity of 3CLpro inhibitors.

## Supplementary Material

Supplementary Tables S1-S3

## Abbreviations

3CLpro: 3-chymotrypsin-like protease
ACE2: angiotensin-converting enzyme 2
AI: artificial intelligence
CoV: coronavirus
COVID-19: coronavirus disease
DMSO: dimethyl sulfoxide
EGFR: epidermal growth factor receptor
EUA: emergency use authorization
FRET: Fluorescence resonance energy transfer
MERS-CoV: Middle East respiratory syndrome coronavirus
ML: machine learning
nsps: nonstructural proteins
orf: open reading frames
PLpro: papin like protease
RdRp: RNA-dependent RNA polymerase
SARS-CoV: severe acute respiratory syndrome coronavirus
