# Supplementary material for: 3-chymotrypsin-like protease in SARS-CoV-2
Source: Biosci Rep. 2024 Aug 5;44(8):BSR20231395. doi: 10.1042/BSR20231395 (PMC11300678; doi:10.1042/BSR20231395)
Supplement: Supplementary Tables S1-S3 [file BSR-2023-1395C_supp.pdf]

Supporting Information

3-chymotrypsin-like protease in SARS-CoV-2

Kenana Al Adem[1,2], Juliana C. Ferreira[1], Adrian J. Villanueva[1], Samar Fadl[1], Farah El-Sadaany[1], Imen Masmoudi[1], Yugmee Gidiya[1], Tariro Gurudza[1], Thyago HS Cardoso[3], Nitin K Saksena[4], Wael M. Rabeh[1*]

[1]Science Division, New York University Abu Dhabi, PO Box 129188, Abu Dhabi, United Arab Emirates.
[2]Institute of Biological and Medical Imaging, Helmholtz Zentrum München, Neuherberg, Germany
[3]OMICS Centre of Excellence, G42 Healthcare, Masdar City, Abu Dhabi, United Arab Emirates
[4]Victoria University, Footscray Campus, Melbourne, VIC. Australia

*Corresponding author: wael.rabeh@nyu.edu

Table S1: Covalent 3CLpro inhibitors. Structures, IC_{50} values, and the PDB codes of co-crystal structures of SARS-CoV-2 3CLpro with covalent inhibitors. The red arrow points to the atom that forms a covalent bond with C145 of 3CLpro. ND: not determined.

**Covalent 3CLpro inhibitors**				
**Peptide-based covalent 3CLpro inhibitors**				
Name	Structure	IC_{50} /EC_{50} (µM)	PDB	Reference
Nirmatrelvir (PF- 07321232)	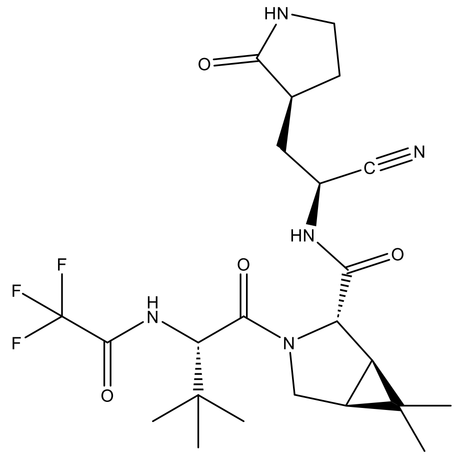	25.7	7SI9	(1, 2)
PF-00835231	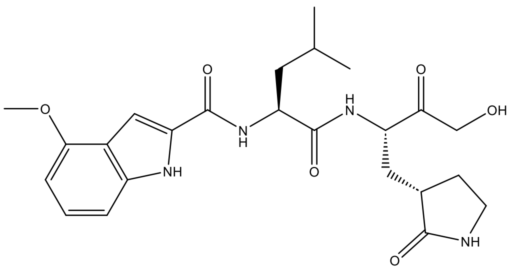	0.23	ND	(3)
Lufotrelvir	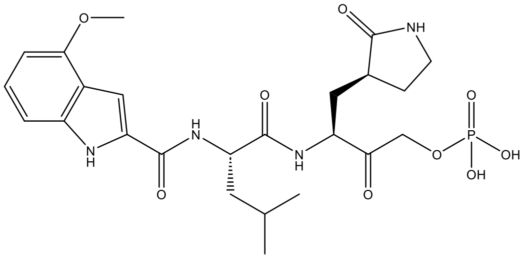	0.69-31.59	7VVP	(4)
Ritonavir	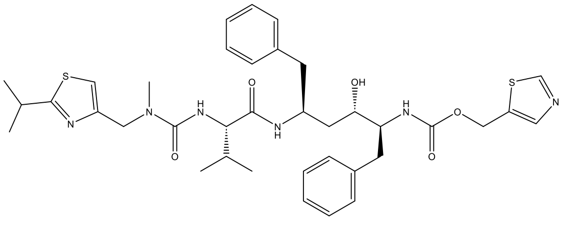	19.88	ND	(5)

Lopinavir	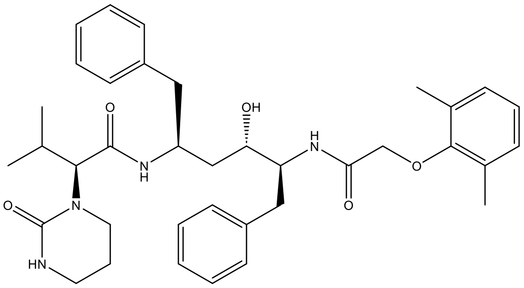	12.01	ND	(5)
GC373	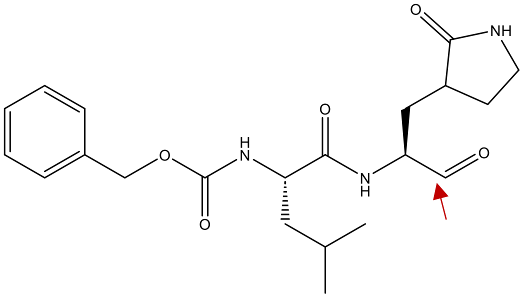	0.4	6WTK	(6-10)
GC376	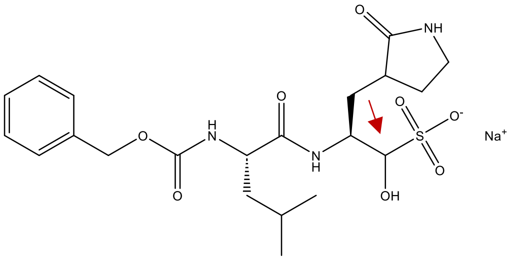	0.19	7D1M/ 6WTJ	(6-10)
Compound 2	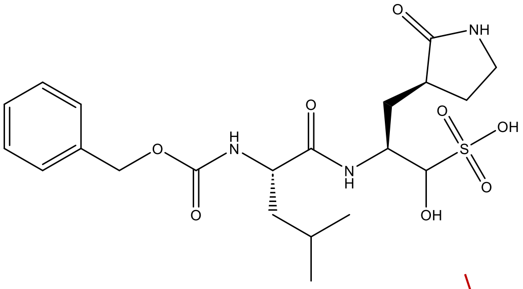	0.18	7K0E	(11)
MPI5	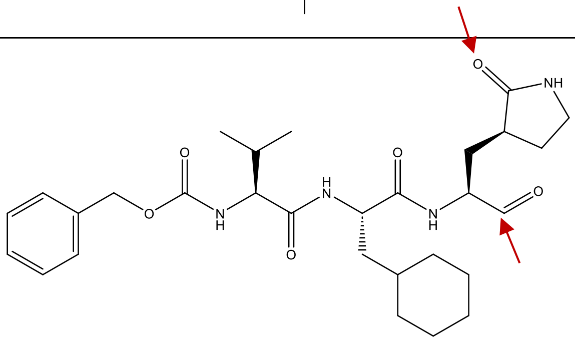	0.033	7JQ2	(12)

MPI8	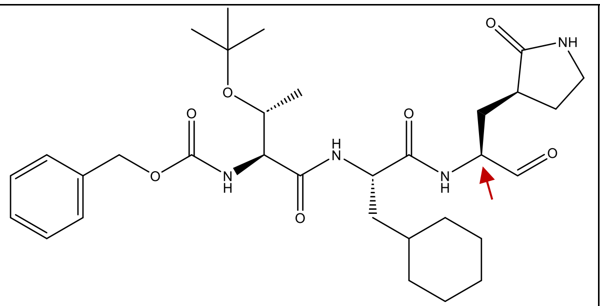	0.105	7JQ5	(12)
Compound 11a	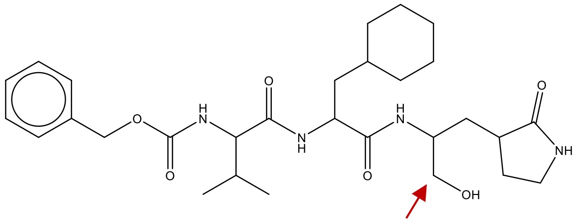	0.031	6LZE	(13)
Compound 11b	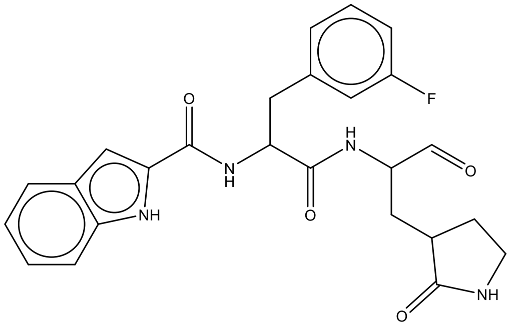	0.04	6M0K	(13)
Compound 2a	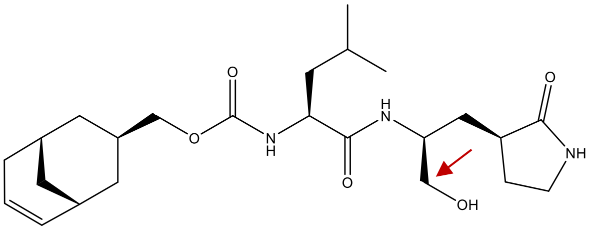	0.18	7LKR	(14)
Compound 13b	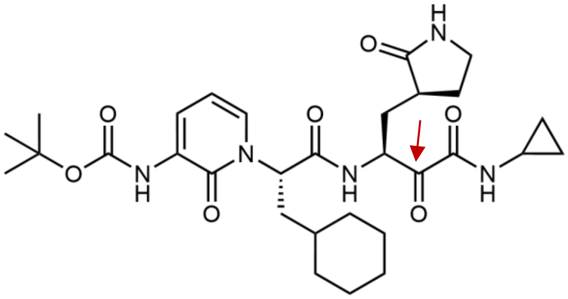	2.39	6Y2G	(15, 16)

AT1001	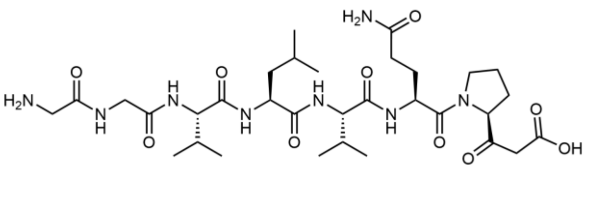	-	ND	(17)
Boceprevir	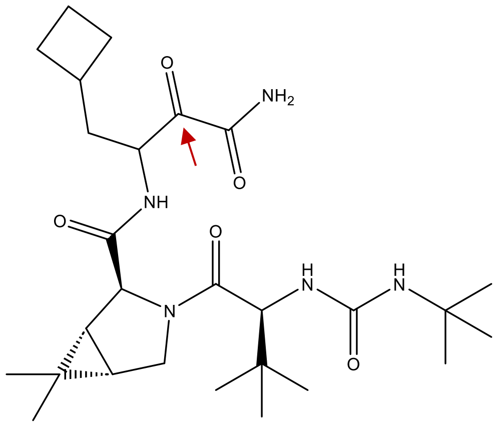	8.0	7C6S	(18, 19)
Simnotrelvir	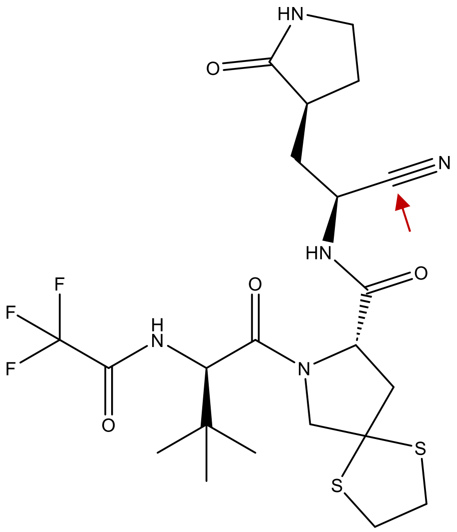	0.008–0.013	8IGX	(20, 21)
RAY1216/ leritrelvir	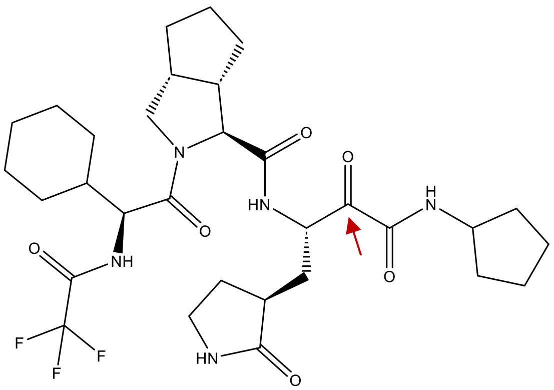	0.095	8IGN	(22)

Tipranavir	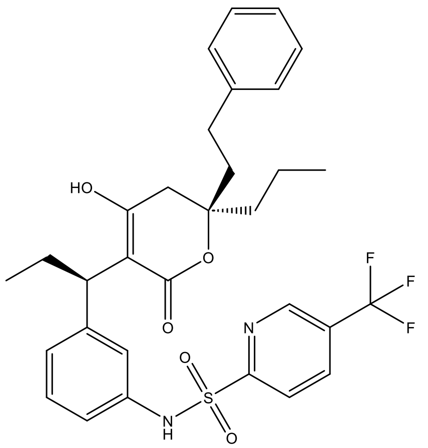	27.6	ND	(23)
Telaprevir	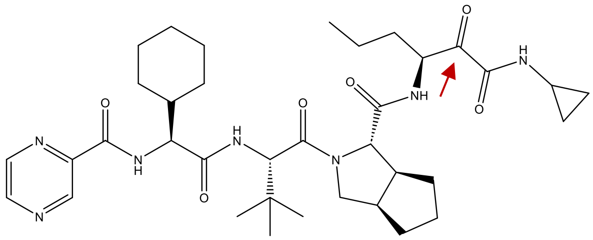	11.6	7K6D	(24)
Narlaprevir	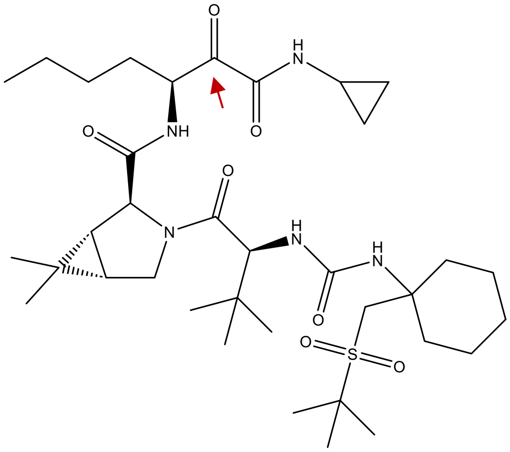	16.11	7JYC	(25)

Paritaprevir	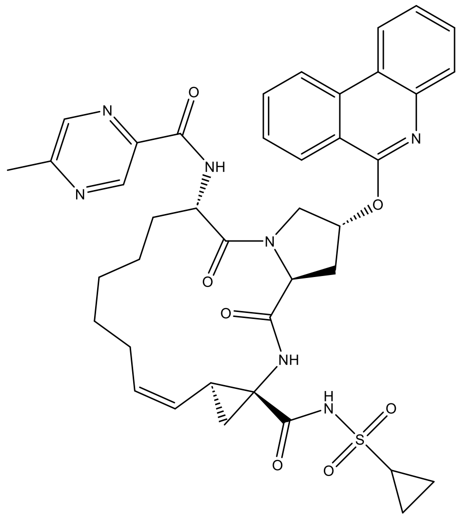	73.38	ND	(23)
Jun9-62-2R	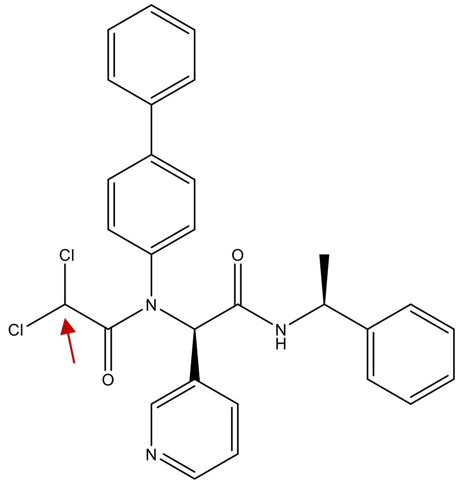	0.43	7RN1	(26)
Jun10541R	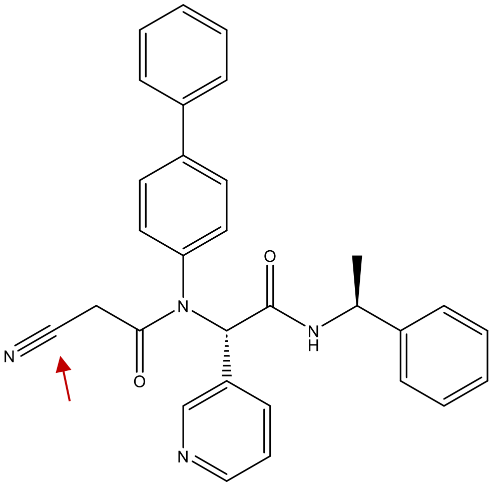	0.50	8FIV	(27)

CDI 45205	Structure not available	-	ND	(28)
AG7404	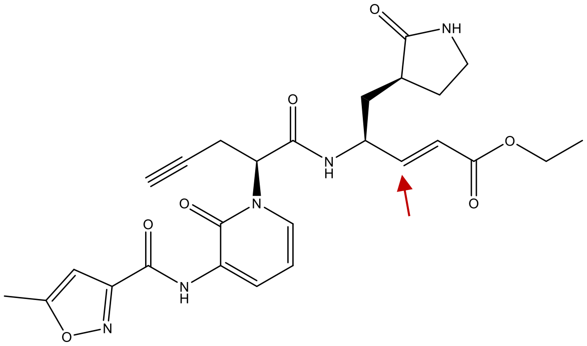	47	7ZQV	(29-31)
AG7088	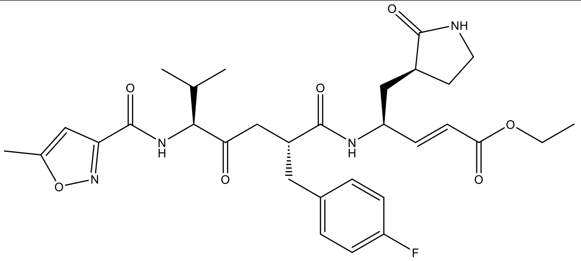	101	ND	(29-31)
**Non-peptide covalent 3CLpro inhibitors**				
Tolperisone (Michael acceptor breakdown product of Tolperisone)	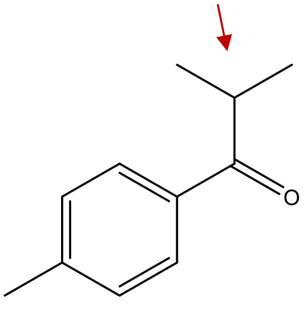	19.17	7ADW	(32)
Ebselen	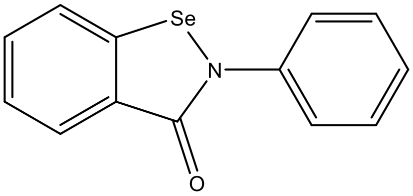	0.67	7BFB	(33)

Carmofur	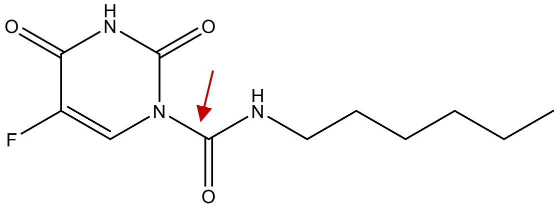	1.82	7BUY	(34)
Disulfiram	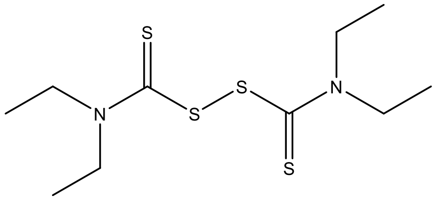	6.25	ND	(33, 35)

Table S2: Non-covalent competitive 3CLpro inhibitors. Structures, IC_{50} values, and the PDB codes of co-crystal structures of SARS-CoV-2 3CLpro with non-covalent competitive inhibitors. These inhibitors target the active site of 3CLpro, where they form non-covalent binding interactions with the active site amino acid residues. ND: not determined.

**Non-covalent 3CLpro inhibitors**				
Name	Structure	IC_{50} (μM)	PDB	Reference
Ensitrelvir	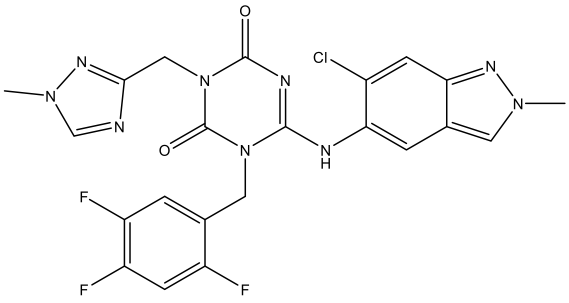	0.049	8DZ0	(36, 37)
Perampanel	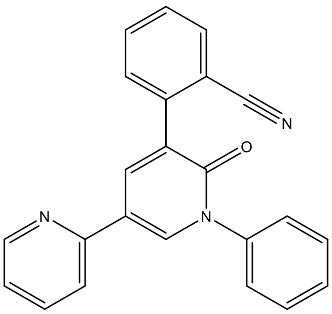	-	ND	(38)
ML188	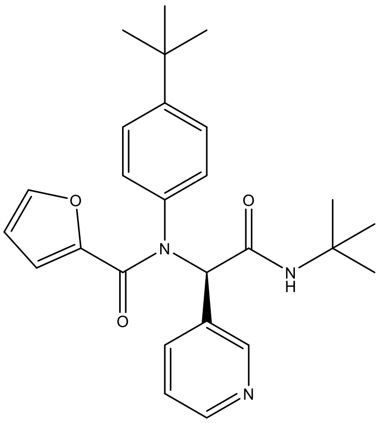	2.5	7L0D	(39-41)

ML300	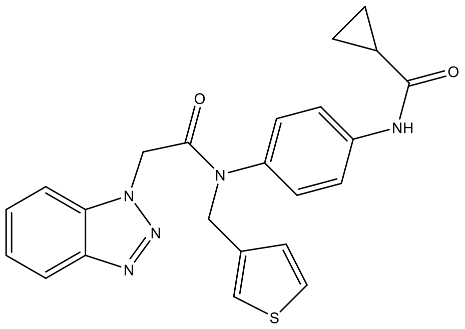	4.9	7LME	(39-41)
23R (Jun8-76-3A)	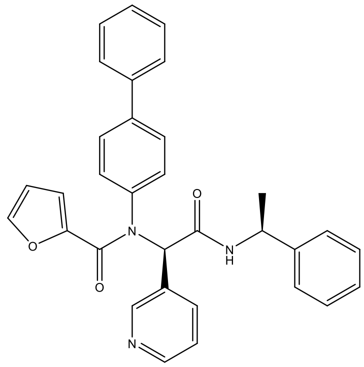	0.20	7KX5	(42)
Masitinib	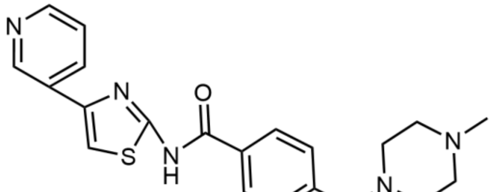	2.5	7JU7	(43)
Baricitinib	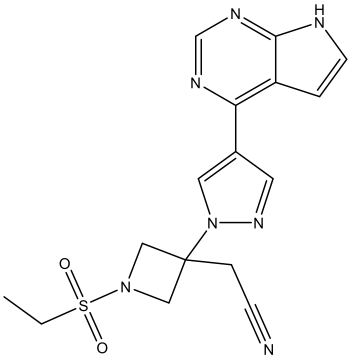	25.3	ND	(44, 45)
Quercetin	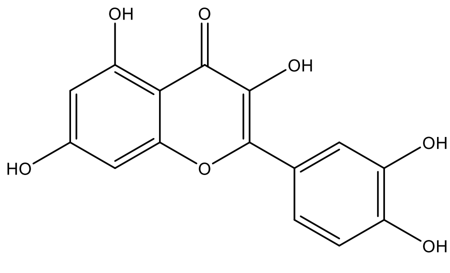	7.40	8GQT	(46)

Resveratrol	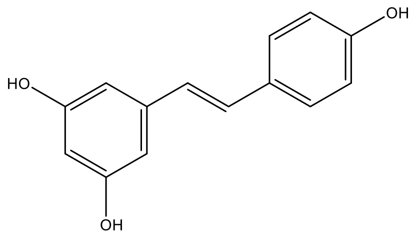	16.9	ND	(47)
Rutin	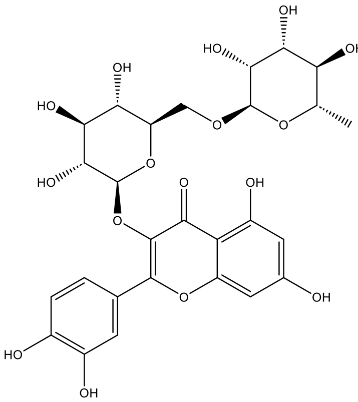	31.3	ND	(48)
Ellagic acid	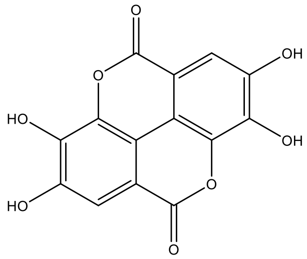	11.8	ND	(47)
Curcumin	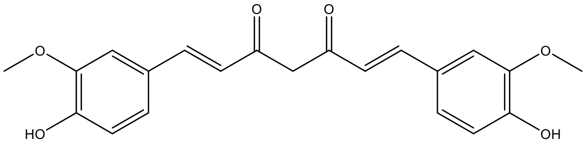	40	ND	(47, 49)
EGCG	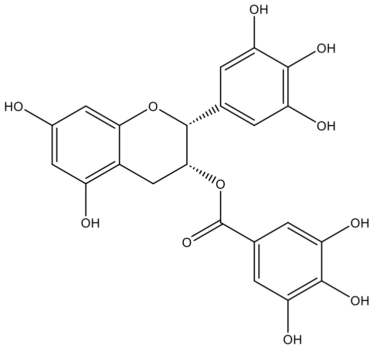	7.5	ND	(50)

Table S3: Non-covalent allosteric 3CLpro inhibitors. Structures, IC_{50} values, and the PDB codes of co-crystal structures of SARS-CoV-2 3CLpro with non-covalent allosteric inhibitors. These inhibitors target allosteric sites of 3CLpro and form non-covalent binding interactions with their residues.

Non-covalent allosteric 3CLpro inhibitors				
Name	Structure	IC_{50} (μM)	PDB	Reference
Pelitinib	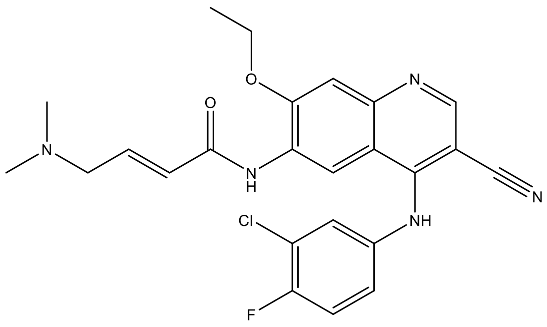	1.25 (EC_{50})	7AXM	(32)
AT7519	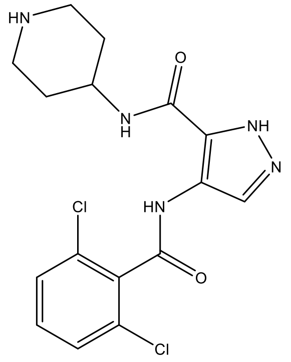	25.16 (EC_{50})	7AGA	(32)
Ifenprodil	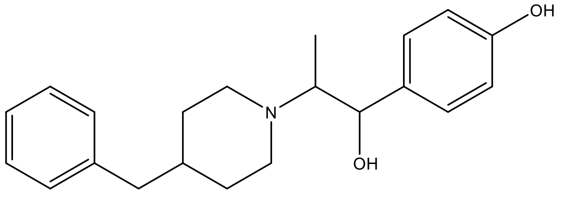	46.86 (EC_{50})	7AQI	(32)

RS-102895	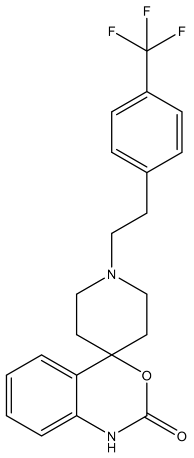	19.8 (EC_{50})	7ABU	(32)
Apixaban	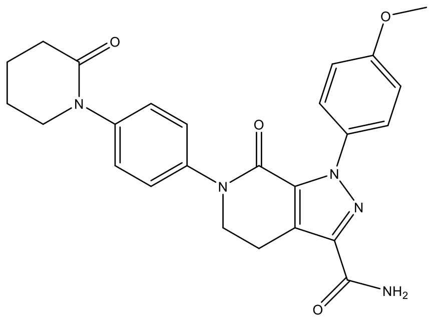	1.49	ND	(51)
Compound 172 (PubChem ID: 3700821)	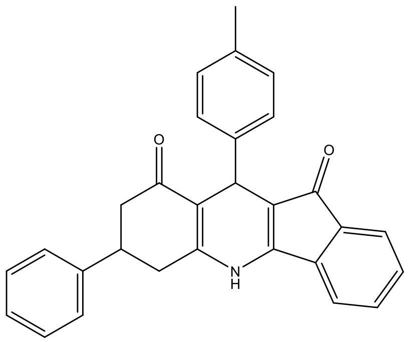	1.82	ND	(52)
Agathisflavone	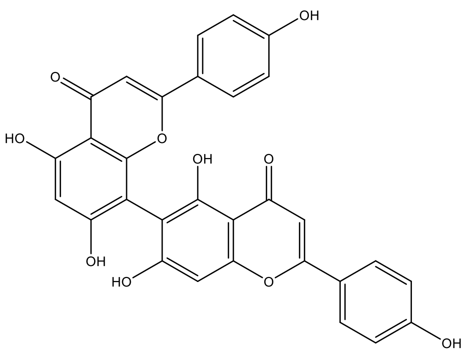	4.32	ND	(53)

References:

1. Owen DR, Allerton CMN, Anderson AS, Aschenbrenner L, Avery M, Berritt S, et al. An oral sars-cov-2 m(pro) inhibitor clinical candidate for the treatment of covid-19. Science. 2021;374(6575):1586-1593. DOI: 10.1126/science.abl4784.
2. Duveau DY, Thomas CJ. The remarkable selectivity of nirmatrelvir. ACS Pharmacol Transl Sci. 2022;5(6):445-447. DOI: 10.1021/acsptsci.2c00065.
3. Boras B, Jones RM, Anson BJ, Arenson D, Aschenbrenner L, Bakowski MA, et al. Preclinical characterization of an intravenous coronavirus 3cl protease inhibitor for the potential treatment of covid19. Nat Commun. 2021;12(1):6055. DOI: 10.1038/s41467-021-26239-2.
4. Li J, Lin C, Zhou X, Zhong F, Zeng P, McCormick PJ, et al. Structural basis of main proteases of coronavirus bound to drug candidate pf-07304814. J Mol Biol. 2022;434(16):167706. DOI: 10.1016/j.jmb.2022.167706.
5. Zhang L, Liu J, Cao R, Xu M, Wu Y, Shang W, et al. Comparative antiviral efficacy of viral protease inhibitors against the novel sars-cov-2 in vitro. Virol Sin. 2020;35(6):776-784. DOI: 10.1007/s12250-020-00288-1.
6. Vuong W, Khan MB, Fischer C, Arutyunova E, Lamer T, Shields J, et al. Feline coronavirus drug inhibits the main protease of sars-cov-2 and blocks virus replication. Nat Commun. 2020;11(1):4282. DOI: 10.1038/s41467-020-18096-2.
7. Arutyunova E, Khan MB, Fischer C, Lu J, Lamer T, Vuong W, et al. N-terminal finger stabilizes the s1 pocket for the reversible feline drug gc376 in the sars-cov-2 m(pro) dimer. J Mol Biol. 2021;433(13):167003. DOI: 10.1016/j.jmb.2021.167003.
8. Liu C, Boland S, Scholle MD, Bardiot D, Marchand A, Chaltin P, et al. Dual inhibition of sars-cov-2 and human rhinovirus with protease inhibitors in clinical development. Antiviral Res. 2021;187:105020. DOI: 10.1016/j.antiviral.2021.105020.
9. Zvornicanin SN, Shaqra AM, Huang QJ, Ornelas E, Moghe M, Knapp M, et al. Crystal structures of inhibitor-bound main protease from delta- and gamma-coronaviruses. Viruses. 2023;15(3). DOI: 10.3390/v15030781.
10. Iketani S, Forouhar F, Liu H, Hong SJ, Lin FY, Nair MS, et al. Lead compounds for the development of sars-cov-2 3cl protease inhibitors. Nat Commun. 2021;12(1):2016. DOI: 10.1038/s41467-021-22362-2.
11. Dampalla CS, Zheng J, Perera KD, Wong LR, Meyerholz DK, Nguyen HN, et al. Postinfection treatment with a protease inhibitor increases survival of mice with a fatal sars-cov-2 infection. Proc Natl Acad Sci U S A. 2021;118(29). DOI: 10.1073/pnas.2101555118.
12. Yang KS, Ma XR, Ma Y, Alugubelli YR, Scott DA, Vatansever EC, et al. A quick route to multiple highly potent sars-cov-2 main protease inhibitors. ChemMedChem. 2021;16(6):942-948. DOI: 10.1002/cmdc.202000924.
13. Dai W, Zhang B, Jiang XM, Su H, Li J, Zhao Y, et al. Structure-based design of antiviral drug candidates targeting the sars-cov-2 main protease. Science. 2020;368(6497):1331-1335. DOI: 10.1126/science.abb4489.
14. Dampalla CS, Kim Y, Bickmeier N, Rathnayake AD, Nguyen HN, Zheng J, et al. Structure-guided design of conformationally constrained cyclohexane inhibitors of severe acute respiratory syndrome coronavirus-2 3cl protease. J Med Chem. 2021;64(14):10047-10058. DOI: 10.1021/acs.jmedchem.1c00319.
15. Zhang L, Lin D, Kusov Y, Nian Y, Ma Q, Wang J, et al. α-ketoamides as broad-spectrum inhibitors of coronavirus and enterovirus replication: Structure-based design, synthesis, and

activity assessment. J Med Chem. 2020;63(9):4562-4578. DOI: 10.1021/acs.jmedchem.9b01828.
16. Zhang L, Lin D, Sun X, Curth U, Drosten C, Sauerhering L, et al. Crystal structure of sars-cov-2 main protease provides a basis for design of improved α-ketoamide inhibitors. Science. 2020;368(6489):409-412. DOI: 10.1126/science.abb3405.
17. Troisi J, Venutolo G, Terracciano C, Carri MD, Di Micco S, Landolfi A, et al. The therapeutic use of the zonulin inhibitor at-1001 (larazotide) for a variety of acute and chronic inflammatory diseases. Curr Med Chem. 2021;28(28):5788-5807. DOI: 10.2174/0929867328666210104110053.
18. Oerlemans R, Ruiz-Moreno AJ, Cong Y, Dinesh Kumar N, Velasco-Velazquez MA, Neochoritis CG, et al. Repurposing the hcv ns3-4a protease drug boceprevir as covid-19 therapeutics. RSC Med Chem. 2020;12(3):370-379. DOI: 10.1039/d0md00367k.
19. Venkatraman S. Discovery of boceprevir, a direct-acting ns3/4a protease inhibitor for treatment of chronic hepatitis c infections. Trends Pharmacol Sci. 2012;33(5):289-294. DOI: 10.1016/j.tips.2012.03.012.
20. Jiang X, Su H, Shang W, Zhou F, Zhang Y, Zhao W, et al. Structure-based development and preclinical evaluation of the sars-cov-2 3c-like protease inhibitor simnotrelvir. Nat Commun. 2023;14(1):6463. DOI: 10.1038/s41467-023-42102-y.
21. Cao B, Wang Y, Lu H, Huang C, Yang Y, Shang L, et al. Oral simnotrelvir for adult patients with mild-to-moderate covid-19. N Engl J Med. 2024;390(3):230-241. DOI: 10.1056/NEJMoa2301425.
22. Chen X, Huang X, Ma Q, Kuzmič P, Zhou B, Xu J, et al. Inhibition mechanism and antiviral activity of an α-ketoamide based sars-cov-2 main protease inhibitor. bioRxiv; 2023.
23. Mody V, Ho J, Wills S, Mawri A, Lawson L, Ebert M, et al. Identification of 3-chymotrypsin like protease (3clpro) inhibitors as potential anti-sars-cov-2 agents. Commun Biol. 2021;4(1):93. DOI: 10.1038/s42003-020-01577-x.
24. Mahmoud A, Mostafa A, Al-Karmalawy AA, Zidan A, Abulkhair HS, Mahmoud SH, et al. Telaprevir is a potential drug for repurposing against sars-cov-2: Computational and in vitro studies. Heliyon. 2021;7(9):e07962. DOI: 10.1016/j.heliyon.2021.e07962.
25. Bai Y, Ye F, Feng Y, Liao H, Song H, Qi J, et al. Structural basis for the inhibition of the sars-cov-2 main protease by the anti-hcv drug narlaprevir. Signal Transduct Target Ther. 2021;6(1):51. DOI: 10.1038/s41392-021-00468-9.
26. Ma C, Xia Z, Sacco MD, Hu Y, Townsend JA, Meng X, et al. Discovery of di- and trihaloacetamides as covalent sars-cov-2 main protease inhibitors with high target specificity. Journal of the American Chemical Society. 2021;143(49):20697-20709. DOI: 10.1021/jacs.1c08060.
27. Tan B, Sacco M, Tan H, Li K, Joyce R, Zhang X, et al. Exploring diverse reactive warheads for the design of sars-cov-2 main protease inhibitors. Eur J Med Chem. 2023;259:115667. DOI: 10.1016/j.ejmech.2023.115667.
28. Vandyck K, Deval J. Considerations for the discovery and development of 3-chymotrypsin-like cysteine protease inhibitors targeting sars-cov-2 infection. Curr Opin Virol. 2021;49:36-40. DOI: 10.1016/j.coviro.2021.04.006.
29. Hayden FG, Turner RB, Gwaltney JM, Chi-Burris K, Gersten M, Hsyu P, et al. Phase ii, randomized, double-blind, placebo-controlled studies of ruprintrivir nasal spray 2-percent suspension for prevention and treatment of experimentally induced rhinovirus colds in

healthy volunteers. Antimicrob Agents Chemother. 2003;47(12):3907-3916. DOI: 10.1128/aac.47.12.3907-3916.2003.

30. Patick AK, Brothers MA, Maldonado F, Binford S, Maldonado O, Fuhrman S, et al. In vitro antiviral activity and single-dose pharmacokinetics in humans of a novel, orally bioavailable inhibitor of human rhinovirus 3c protease. Antimicrob Agents Chemother. 2005;49(6):2267-2275. DOI: 10.1128/aac.49.6.2267-2275.2005.
31. Fàbrega-Ferrer M, Herrera-Morandé A, Muriel-Goñi S, Pérez-Saavedra J, Bueno P, Castro V, et al. Structure and inhibition of sars-cov-1 and sars-cov-2 main proteases by oral antiviral compound ag7404. Antiviral Res. 2022;208:105458. DOI: 10.1016/j.antiviral.2022.105458.
32. Günther S, Reinke PYA, Fernández-García Y, Lieske J, Lane TJ, Ginn HM, et al. X-ray screening identifies active site and allosteric inhibitors of sars-cov-2 main protease. Science. 2021;372(6542):642-646. DOI: 10.1126/science.abf7945.
33. Ma C, Hu Y, Townsend JA, Lagarias PI, Marty MT, Kolocouris A, et al. Ebselen, disulfiram, carmofur, px-12, tideglusib, and shikonin are nonspecific promiscuous sars-cov-2 main protease inhibitors. ACS Pharmacol Transl Sci. 2020;3(6):1265-1277. DOI: 10.1021/acsptsci.0c00130.
34. Jin Z, Zhao Y, Sun Y, Zhang B, Wang H, Wu Y, et al. Structural basis for the inhibition of sars-cov-2 main protease by antineoplastic drug carmofur. Nat Struct Mol Biol. 2020;27(6):529-532. DOI: 10.1038/s41594-020-0440-6.
35. Jin Z, Du X, Xu Y, Deng Y, Liu M, Zhao Y, et al. Structure of m(pro) from sars-cov-2 and discovery of its inhibitors. Nature. 2020;582(7811):289-293. DOI: 10.1038/s41586-020-2223-y.
36. Unoh Y, Uehara S, Nakahara K, Nobori H, Yamatsu Y, Yamamoto S, et al. Discovery of s-217622, a noncovalent oral sars-cov-2 3cl protease inhibitor clinical candidate for treating covid-19. J Med Chem. 2022;65(9):6499-6512. DOI: 10.1021/acs.jmedchem.2c00117.
37. Lin M, Zeng X, Duan Y, Yang Z, Ma Y, Yang H, et al. Molecular mechanism of ensitrelvir inhibiting sars-cov-2 main protease and its variants. Commun Biol. 2023;6(1):694. DOI: 10.1038/s42003-023-05071-y.
38. Deshmukh MG, Ippolito JA, Zhang C-H, Stone EA, Reilly RA, Miller SJ, et al. Structure-guided design of a perampanel-derived pharmacophore targeting the sars-cov-2 main protease. Structure. 2021;29(8):823-833.e825. DOI: https://doi.org/10.1016/j.str.2021.06.002.
39. Han SH, Goins CM, Arya T, Shin WJ, Maw J, Hooper A, et al. Structure-based optimization of ml300-derived, noncovalent inhibitors targeting the severe acute respiratory syndrome coronavirus 3cl protease (sars-cov-2 3cl(pro)). J Med Chem. 2022;65(4):2880-2904. DOI: 10.1021/acs.jmedchem.1c00598.
40. Jacobs J, Grum-Tokars V, Zhou Y, Turlington M, Saldanha SA, Chase P, et al. Discovery, synthesis, and structure-based optimization of a series of n-(tert-butyl)-2-(n-arylamido)-2-(pyridin-3-yl) acetamides (ml188) as potent noncovalent small molecule inhibitors of the severe acute respiratory syndrome coronavirus (sars-cov) 3cl protease. J Med Chem. 2013;56(2):534-546. DOI: 10.1021/jm301580n.
41. Lockbaum GJ, Reyes AC, Lee JM, Tilvawala R, Nalivaika EA, Ali A, et al. Crystal structure of sars-cov-2 main protease in complex with the non-covalent inhibitor ml188. Viruses. 2021;13(2). DOI: 10.3390/v13020174.

42. Kitamura N, Sacco MD, Ma C, Hu Y, Townsend JA, Meng X, et al. Expedited approach toward the rational design of noncovalent sars-cov-2 main protease inhibitors. J Med Chem. 2022;65(4):2848-2865. DOI: 10.1021/acs.jmedchem.1c00509.
43. Drayman N, DeMarco JK, Jones KA, Azizi SA, Froggatt HM, Tan K, et al. Masitinib is a broad coronavirus 3cl inhibitor that blocks replication of sars-cov-2. Science. 2021;373(6557):931-936. DOI: 10.1126/science.abg5827.
44. Anton DB, Galvez Bulhões Pedreira J, Zvirtes ML, Laufer SA, Ducati RG, Goettert M, et al. Targeting sars-cov-2 main protease (mpro) with kinase inhibitors: A promising approach for discovering antiviral and anti-inflammatory molecules against sars-cov-2. J Chem Inf Model. 2023;63(13):4138-4146. DOI: 10.1021/acs.jcim.3c00324.
45. Hojjat-Farsangi M. Small-molecule inhibitors of the receptor tyrosine kinases: Promising tools for targeted cancer therapies. Int J Mol Sci. 2014;15(8):13768-13801. DOI: 10.3390/ijms150813768.
46. Abian O, Ortega-Alarcon D, Jimenez-Alesanco A, Ceballos-Laita L, Vega S, Reyburn HT, et al. Structural stability of sars-cov-2 3clpro and identification of quercetin as an inhibitor by experimental screening. Int J Biol Macromol. 2020;164:1693-1703. DOI: 10.1016/j.ijbiomac.2020.07.235.
47. Bahun M, Jukić M, Oblak D, Kranjc L, Bajc G, Butala M, et al. Inhibition of the sars-cov-2 3cl(pro) main protease by plant polyphenols. Food Chem. 2022;373(Pt B):131594. DOI: 10.1016/j.foodchem.2021.131594.
48. Liao Q, Chen Z, Tao Y, Zhang B, Wu X, Yang L, et al. An integrated method for optimized identification of effective natural inhibitors against sars-cov-2 3clpro. Sci Rep. 2021;11(1):22796. DOI: 10.1038/s41598-021-02266-3.
49. Marín-Palma D, Tabares-Guevara JH, Zapata-Cardona MI, Flórez-Álvarez L, Yepes LM, Rugeles MT, et al. Curcumin inhibits in vitro sars-cov-2 infection in vero e6 cells through multiple antiviral mechanisms. Molecules. 2021;26(22). DOI: 10.3390/molecules26226900.
50. Jang M, Park YI, Cha YE, Park R, Namkoong S, Lee JI, et al. Tea polyphenols egcg and theaflavin inhibit the activity of sars-cov-2 3cl-protease in vitro. Evid Based Complement Alternat Med. 2020;2020:5630838. DOI: 10.1155/2020/5630838.
51. Chaves OA, Sacramento CQ, Fintelman-Rodrigues N, Temerozo JR, Pereira-Dutra F, Mizurini DM, et al. Apixaban, an orally available anticoagulant, inhibits sars-cov-2 replication and its major protease in a non-competitive way. J Mol Cell Biol. 2022;14(6). DOI: 10.1093/jmcb/mjac039.
52. Chan CC-Y, Guo Q, Chan JF-W, Tang K, Cai J-P, Chik KK-H, et al. Identification of novel small-molecule inhibitors of sars-cov-2 by chemical genetics. Acta Pharmaceutica Sinica B. 2024. DOI: https://doi.org/10.1016/j.apsb.2024.05.026.
53. Chaves OA, Lima CR, Fintelman-Rodrigues N, Sacramento CQ, de Freitas CS, Vazquez L, et al. Agathisflavone, a natural biflavonoid that inhibits sars-cov-2 replication by targeting its proteases. Int J Biol Macromol. 2022;222:1015-1026. DOI: https://doi.org/10.1016/j.ijbiomac.2022.09.204.
